# Visual Impairment and Suicide Risk: A Systematic Review and Meta-Analysis

**DOI:** 10.1001/jamanetworkopen.2024.7026

**Published:** 2024-04-17

**Authors:** Chung Young Kim, Ahnul Ha, Sung Ryul Shim, In Hwan Hong, In Boem Chang, Young Kook Kim

**Affiliations:** 1 Department of Ophthalmology, Seoul National University Hospital, Seoul, Korea; 2 Department of Ophthalmology, Seoul National University College of Medicine, Seoul, Korea; 3 Department of Ophthalmology, Jeju National University Hospital, Jeju-si, Korea; 4 Department of Ophthalmology, Jeju National University College of Medicine, Jeju-si, Korea; 5 Department of Biomedical Informatics, College of Medicine, Konyang University, Daejeon, Korea; 6 Department of Ophthalmology, Dongtan Sacred Heart Hospital, Hwaseong, Korea; 7 Hallym University Medical Center, Hwaseong, Korea; 8 Seoul ON Eye Clinic, Seoul, Korea; 9 EyeLight Data Science Laboratory, Seoul National University College of Medicine, Seoul, Korea; 10 Trinity Biomedical Sciences Institute, Trinity College Dublin, Dublin, Ireland

## Abstract

**Importance:**

Suicide is a substantial public health concern that involves various recognized contributing factors. Sensory impairments, specifically visual impairment, are deemed potential risk factors. Nonetheless, comprehensive information about associated risk levels and underlying determinants remains limited.

**Objective:**

To investigate the association between visual impairment and different aspects of suicide, including the assessment of risk levels and exploration of potential contributing factors.

**Data Sources:**

An electronic search was performed in the PubMed, EMBASE, Scopus, and Cochrane Library databases from their inception to February 8, 2024.

**Study Selection:**

All published studies were considered without restrictions on study design, publication date, or language.

**Data Extraction and Synthesis:**

Two independent reviewers extracted the published data using a standardized procedure in accordance with the Meta-analysis of Observational Studies in Epidemiology (MOOSE) and Preferred Reporting Items for Systematic Reviews and Meta-analyses (PRISMA) reporting guidelines. Random-effects meta-analyses were used to estimate pooled effect sizes. Multiple meta-regression analyses were conducted to identify potential factors contributing to the association between visual impairment and the risk of suicide.

**Main Outcomes and Measures:**

The primary outcome measure was the odds ratio (OR) of suicidal behavior (including suicide attempt and suicide death) for individuals with visual impairment compared with those without. The secondary outcome measures were the pooled ORs of suicidal ideation and suicide death, respectively.

**Results:**

A total of 31 population-based studies with 5 692 769 unique individuals (mean [SD] age, 48.4 [8.5] years; 2 965 933 females [52%]) were included. For 17 studies (5 602 285 individuals) that evaluated suicidal behavior, the pooled OR was 2.49 (95% CI, 1.71-3.63). For 21 studies (611 899 individuals) that assessed suicidal ideation, the pooled OR was 2.01 (95% CI, 1.62-2.50). For 8 studies (5 067 113 individuals) investigating the association between visual impairment and suicide death, the pooled OR was 1.89 (95% CI, 1.32-2.71). The multiple meta-regression model identified age group as a predictive factor associated with suicidal behavior, with the studies included suggesting that adolescents were at the highest risk. While this analysis showed moderate heterogeneity for suicide death, high heterogeneity was observed for suicidal behavior and suicidal ideation.

**Conclusions and Relevance:**

The findings of this systematic review and meta-analysis support the association between visual impairment and increased risk of suicidal tendencies. The risk differed by age group, with a pronounced risk observed among adolescents.

## Key Points

**Question:**

Is visual impairment associated with suicide risk, and if so, what factors contribute to the association?

**Findings:**

In this systematic review and meta-analysis including 31 studies and 5 692 769 unique individuals, visual impairment was associated with an increased risk of suicide, including suicidal ideation, suicidal behavior, and suicide death. This elevated risk was particularly pronounced among adolescents with visual impairment.

**Meaning:**

These findings suggest an association between visual impairment and an elevated risk of suicidal tendencies, with variations in risk observed across age groups and a particularly pronounced risk among adolescents.

## Teaser

This systematic review and meta-analysis investigates the association between visual impairment and suicidal behavior and explores potential factors moderating the association.

## Introduction

Suicide, which has an estimated annual death toll of nearly 1 million lives worldwide,^1^ is a significant and urgent global public health challenge. Although a growing body of literature has addressed the topic of suicide, its comprehension and synthesis can be complex due to the various phenotypes that fall within the spectrum of suicide. These phenotypes encompass suicidal ideation, which is characterized by thoughts of ending one’s own life in an active form (with a specific plan) or in a passive form (with a mere desire to die but lacking a concrete plan), suicide attempt, and death by suicide.^2^

Risk factors for suicidal ideation differ from those for the transition to suicidal behavior, which includes suicide attempt or completed suicide.^3^ Family history of suicide or suicide attempt, mental disorders, chronic physical illness, and sociodemographic factors contribute to increased risk of suicide.^4,5^ Among older populations, additional risk factors include sleep disorders, reduced mobility, compromised quality of life, and significant functional impairment.^6,7^

More than 500 million individuals are blind or have significant visual impairment worldwide.^8^ Visual impairment is linked to challenges such as decreased independence, social skills, and personal income.^9^ It also raises the risk of mental health issues such as depression, stress, and a decline in overall quality of life.^10^ Accordingly, previous studies have suggested a plausible association between visual impairment and increased risk of suicide.^4,11,12^ Nonetheless, the consistency and magnitude of this association exhibit variability among studies, posing a challenge for assessments of the precise nature of the association and the extent of the associated risk. The objective of this systematic review and meta-analysis was to consolidate the available literature on the association between visual impairment and diverse aspects of suicide, with the intention of illuminating both the extent of this association and the potential risk factors.

## Methods

This study was exempt from institutional review board approval and the need for informed consent, as it exclusively used previously published data and did not qualify as human participant research according to the Seoul National University Hospital Institutional Review Board guidelines. The study followed the Meta-Analysis of Observational Studies in Epidemiology (MOOSE) and Preferred Reporting Items for Systematic Reviews and Meta-Analyses (PRISMA) reporting guidelines. The study protocol was prospectively registered with PROSPERO (CRD42022325106) and has been published.^13^

### Search Strategy and Selection Criteria

With the assistance of an academic librarian, we conducted a systematic literature search of key databases, including PubMed, EMBASE, Scopus, and the Cochrane Library, to identify relevant studies from their inception to February 8, 2024. Our search strategy used a combination of medical subject headings and text words related to visual impairment and suicide. The search terms encompassed concepts such as *visual impairment*, *low vision*, *blindness*, *suicide*, *suicidal ideation*, *suicide attempt*, *suicidal behavior*, and *association.* No restrictions were imposed in terms of the study design, publication date, or language. Additionally, we manually searched the reference lists of published articles to identify any relevant studies missed in the electronic searches. The complete search strategy is outlined in eAppendix 1 in Supplement 1.

Two independent reviewers (C.Y.K. and A.H.) rigorously screened the titles and abstracts of the identified studies in accordance with predefined inclusion criteria. Subsequently, the full-text articles of the potentially eligible studies underwent meticulous evaluation for inclusion by the reviewers. Any discrepancies or disagreements during the screening process were resolved through consultation with a third investigator (Y.K.K.). In cases where multiple publications reported findings from the same study population, we included only the most comprehensive report with the largest sample size after verifying for duplicates.

We included studies that met the following criteria: (1) population-based; (2) reporting visual impairment as a covariate; (3) incorporating suicide death, suicidal ideation, or suicide attempts as outcome measures; and (4) providing odds ratios (ORs) or relative risks (RRs) with corresponding 95% CIs as measures of association or allowing for computation of these measures based on count data reported in the article. We excluded studies that (1) focused solely on pediatric populations; (2) constituted narrative and/or systematic reviews, case reports, commentaries, editorials, or conference abstracts; and (3) lacked a clear definition of visual impairment or a detailed description of the assessment of suicide.

### Data Extraction

For each included study, data extraction was performed independently by 2 reviewers (C.Y.K. and A.H.) using a standardized data collection form available in Microsoft Access 2016 (Microsoft Corp). Conflicting data entries were identified by algorithm. The extracted information was as follows: (1) study identification details, such as name of first author and publication year; (2) baseline study year; (3) country of study; (4) total number of participants; (5) race and ethnicity of participants, since disparities in suicide rates among populations based on race and ethnicity are well known; (6) age and sex distribution of participants; (7) details on visual impairment assessment; (8) aspects of suicide measured; (9) measures of association (ORs or RRs) with accompanying 95% CIs; and (10) adjustment for confounding factors, if applicable.

Regarding visual impairment, we extracted information pertaining to (1) the operational definition of visual impairment used in the study and (2) the specific method used for visual impairment assessment. For suicide-related outcomes, we extracted (1) the precise definitions of suicidal ideation and suicide attempts as stipulated within the study and (2) the method used to confirm instances of suicide death. In instances where specific details were not readily available within the published article, attempts were made to contact the corresponding author and solicit supplementary information.

### Statistical Analysis

The primary outcome measure was the pooled OR of suicidal behavior among individuals with visual impairment compared with those without, and the secondary outcome measures were the pooled ORs of suicidal ideation and suicide death. We conducted a meta-analysis for exposure (visual impairment) and each outcome combination (suicidal behavior, suicidal ideation, and suicide death) using inverse variance–weighted random-effects models to combine the study-specific measures of association. We included both unadjusted and adjusted estimates of increased risk, giving priority to adjusted estimates for our analysis. When count data were available, we calculated unadjusted measures of association. In cases where neither count data nor risk estimates were provided, we calculated the standardized mortality ratio based on the reference-population statistics provided in the respective studies.^14^ The quantification of between-study outcome variation (ie, heterogeneity) was conducted using the *I*^2^ statistic. This metric illustrates the percentage of variation across studies attributed to heterogeneity rather than to chance, irrespective of the treatment effect metric used.^15^ Drapery plots were created to illustrate, as curves, the *P* value function for each individual study and pooled estimates in each meta-analysis, along with the prediction range for a single future study.^16^

Due to the discrepancies in definitions of the degree of visual impairment (which included both low vision and blindness)^17^ across studies, it was impractical to combine multiple study findings by visual impairment severity. Consequently, we consolidated multiple ORs provided by a study based on visual impairment severity categories to derive a single OR value per study. In cases where individual studies presented different ratio measures of association (eg, OR and/or RR), we considered these estimates to be reasonably similar given the rarity of suicide occurrences.^18^

We also conducted meta-regression analyses to investigate possible causes of heterogeneity across studies.^19^ These analyses aimed to explore differences in study characteristics and populations that potentially could have altered the association with visual impairment on the risk of suicide. The 8 covariates included were as follows: (1) publication year, (2) main ethnicity of study participants, (3) mean (SD) age of study participants, (4) total sample size, (5) vision assessment method, (6) consideration of potential confounding factors, (7) continents where study was conducted, and (8) studies conducted in low-income country. Definitions of each of the covariates are provided in eTable 1 in Supplement 1.

In multiple meta-regression analysis, one can try to model all possible combinations of predictive factors associated with outcomes (ie, covariates) in a procedure termed *multimodel inference*. This allows for determination of which possible covariate combination provides the best fit and which are the most important overall.^20^ We modeled all possible combinations of 8 covariates (2^8^ = 256) and determined the model that performed best with a lowest value of Akaike information criterion corrected.

Additionally, graphic display of heterogeneity (GOSH) plot analysis,^21^ a sophisticated means of exploring the patterns of effect sizes and heterogeneity in our data, was performed. This method uses 3 clustering algorithms: *K*-means clustering,^22^ density-based spatial clustering of applications with noise,^23^ and Gaussian mixture models.^24^ For those plots, we fit the same meta-analysis model to all possible subsets of our included studies. Then, sensitivity analysis was applied to test the effect of rerunning the meta-analysis after removing studies potentially contributing to cluster imbalance. All statistical analyses were conducted using R, version 4.0.4 (R Project for Statistical Computing). Two-sided *P* < .05 was considered statistically significant.

To assess the methodological quality of the included studies, we used the Newcastle-Ottawa Scale, a validated tool for evaluating the quality of cross-sectional, case-control, and cohort studies (eAppendix 2 in Supplement 1).^25^ Our qualitative assessment for publication bias involved the use of funnel plots.^26^

## Results

### Study Selection and Appraisal

**Figure 1.**
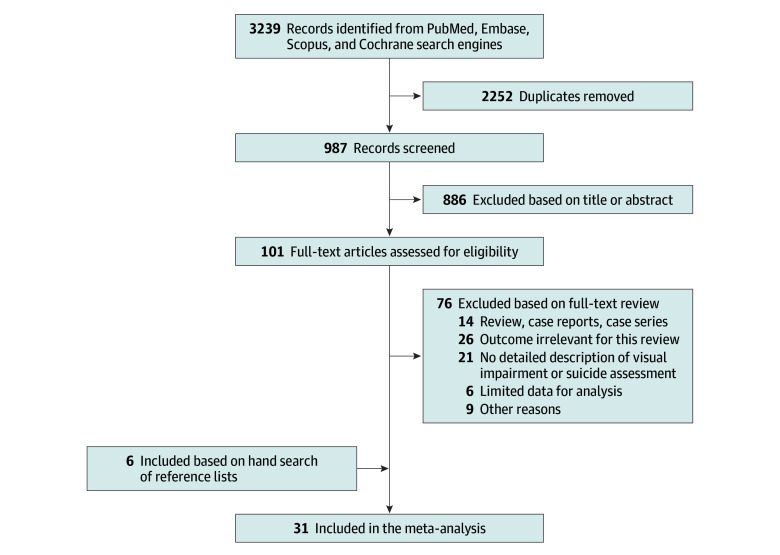
Study Flow Diagram

A total of 3239 studies were initially identified through a systematic search. Following a process of duplicate exclusion and abstract screening, 101 studies were considered potentially relevant and subjected to a thorough full-text review. Ultimately, 31 studies were included,^27,28,29,30,31,32,33,34,35,36,37,38,39,40,41,42,43,44,45,46,47,48,49,50,51,52,53,54,55,56,57^ encompassing a combined study population of 5 692 769 individuals (mean [SD] age, 48.4 [8.5] years; 2 965 933 female [52%] and 2 726 836 male [48%]). The stepwise selection process is depicted in Figure 1.

The publication timeline of the studies varied, with 2 studies published in the 1990s,^27,28^ 5 in the 2000s,^29,30,31,32,33^ 12 in the 2010s,^34,36,37,38,39,40,41,42,43,44,45,46^ and 12 in the 2020s.^35,47,48,49,50,51,52,53,54,55,56,57^ Geographically, the studies covered a diverse range of regions, with 8 studies conducted in Europe,^28,29,30,33,38,44,45,50^ 10 in Australia and Asia,^27,31,39,40,41,42,43,46,56,57^ 11 in North America,^32,34,36,37,47,48,51,52,53,54,55^ 1 in the Middle East,^35^ and 1 involving multiple countries.^49^ The detailed characteristics of each included study can be found in Table 1. The definitions and criteria of the methods used to assess visual impairment and risk of suicide are provided in Table 2.

**Table 1.** Study Characteristics

Source	Study design	Country/ethnicity and race	Study population	Sample size	No. with visual impairment (No. with suicidality)	OR (95% CI)	Adjusted confounders
Jorm et al,^27^ 1995	Cross-sectional	Australia/Australian	Adults aged ≥70 y	923	44 (8 SI)	9.4 (3.4-26.0) (SI)	Depressive symptoms
Forsell et al,^28^ 1997	Cross-sectional	Sweden/Swedish	Adults aged ≥75 y	969	426 (133 SI)	1.61 (1.29-2.01) (SI)	NA
Waern et al,^29^ 2002	Case-control	Sweden/Swedish	Adults aged ≥65 y	238	18 (12 S)	7.0 (2.3-21.4) (S)	NA
Waern et al,^30^ 2003	Case-control	Sweden/Swedish	Adults aged ≥65 y	238	18 (12 S)	6.29 (1.92-20.59) (S)	Age
Yip et al,^31^ 2003	Cross-sectional	Hong Kong/Chinese	Adults aged ≥60 y	917	NA	3.34 (1.24-9.04) (SI)	NA
Mojon-Azzi et al,^33^ 2008	Cross-sectional	10 EU countries/multiple	Adults aged ≥50 y	22 486	717 (144 SI)	3.51 (3.00-4.10) (SI)	Age, sex, country, and cohabitant status
Lam et al,^32^ 2008	Cross-sectional	US/multiple	Adults aged ≥18 y	137 479	18 (200 S)	1.50 (0.90-2.49) (S)	Age, sex, race, marital status, No. of nonocular health conditions, self-rated health, survey design
Fässberg et al,^45^ 2013	Cross-sectional	Sweden/Swedish	Adults aged 97 y	269	77 (8 SI)	0.87 (1.22-36.86) (SI)	NA
Okamura et al,^41^ 2014	Cross-sectional	Japan/Japanese	Homeless individuals in Tokyo	423	166 (18 SI)	1.79 (0.79-4.06) (SI)	Depression
Cho et al,^42^ 2015	Cross-sectional	South Korea/Korean	Adults aged ≥19 y	28 392	166 (NA SI)	2.10 (1.38-3.19) (SI)	Age, sex
Kim et al,^39^ 2015	Cross-sectional	South Korea/Korean	Adults aged ≥65 y	3636	910 (244 SI)	1.65 (1.16-2.35) (SI)	Age, sex, marital status, economic status, smoking, diabetes, osteoarthritis, stroke, heart disease, dizziness, fall, experience of depression
Rim et al,^40^ 2015	Cross-sectional	South Korea/Korean	Adults aged ≥19 y	28 919	10 961 (3671 SI; 14 SA)	1.85 (1.04-3.27) (SI); 3.44 (0.92-12.79) (SA)	Age, sex, household
Meyer-Rochow et al,^44^ 2015	Cohort	Finland/Finnish	Adults aged ≥70 y	91	91 (91 S)	1.31 (1.07-1.61) (S)	Income, educational level, occupation, residential area, spouse, alcohol use
Jung and Park,^43^ 2016	Cross-sectional	South Korea/Korean	Adults aged ≥40 y	12 079	570 with glaucoma (108 SI)	1.30 (1.09-1.55) (SI)	NA
Morton,^37^ 2017	Cross-sectional	US/Swedish	Swedish twins	43 703	2018 (77 S)	0.96 (0.41-2.55) (S)	NA
Moses,^34^ 2018	Cross-sectional	US/multiple	Adolescents	13 362	376 (52 SA)	6.77 (5.08-9.00) (SA)	NA
Alvarado-Esquivel,^36^ 2018	Cross-sectional	Mexico/Mestizos	Middle-aged women	395	205 (20 SA)	1.72 (0.70-4.22) (SA)	NA
Cosh et al,^38^ 2019	Cohort	France/French	Adults aged ≥73 y	5438	325 (44 SI)	1.59 (1.06-2.38) (SI)	Age, sex, center
Na et al,^46^ 2019	Cross-sectional	South Korea/Korean	All age groups	59 596	233 (17 S)	2.87 (1.52-5.43) (S)	Age, income level, disability, psychiatric disorders, physical disorders
Akram and Batool,^35^ 2020	Cross-sectional	Pakistan/Punjabi	Adolescents and young adults	1132	537 (470 SI; 178 SA)	1.55 (1.34-1.78) (SI); 15.5 (8.71-27.59) (SA)	NA
Park and Lee,^47^ 2020	Cross-sectional	US/multiple	Adults aged ≥65 y	10 635	729 (34 SI)	3.12 (1.24-7.85) (SI)	Sex, educational level, race, employment status, marital status, poverty, urbanization, health status, depression, alcohol use, cigarette smoking
Smith et al,^48^ 2020	Cross-sectional	US/multiple	Adults aged ≥60 y with vision-related diagnoses	101	101 (6 SI)	2.28 (0.46-4.11) (SI)	NA
Khurana et al,^50^ 2021	Cross-sectional	England/multiple	Adults aged ≥16 y	7546	1028 (89 SI; 18 SA)	2.05 (1.51-2.78) (SI); 4.97 (2.37-10.41) (SA)	Age, sex, ethnicity, social deprivation, diabetes
Okoro et al,^51^ 2021	Cross-sectional	US/multiple	Adults aged ≥18 y	1004	26 (13 SI)	6.69 (4.30-10.43) (SI)	NA
Parker et al,^52^ 2021	Cross-sectional	US/multiple	University students	3212	167 (NA SI)	3.03 (1.92-4.78) (SI)	Age, birth sex, race, income
Marlow et al,^53^ 2021	Cross-sectional	US/multiple	Adults aged ≥18 y	198 640	3080 (114 SI; 22 SA)	1.61 (1.27-2.04) (SI); 1.65 (1.14-2.38) (SA)	Age, sex, race and ethnicity, marital status, educational level, employment, income, insurance, smoking, drinking, obesity, health status, No. of emergency visits, No. of comorbidities, depressive episode
Marlow et al,^54^ 2022	Cross-sectional	US/multiple	Adults aged ≥18 y	36 544	NA	0.99 (0.71-1.38) (SI); 1.27 (0.60-2.67) (SA)	Age, sex, race and ethnicity, marital status, educational level, employment status, household income, insurance status, urbanization, smoking status, alcohol dependence, illicit drug dependence, health status, No. of ED visits, No. of comorbidities, depressive episode, year of survey
Smith et al,^49^ 2022	Cross-sectional	Multiple/multiple	Adults aged ≥50 y	34 129	5392 (1160 SI; 205 SA)	2.72 (0.18-40.77) (SI); 2.39 (0.02-263.55) (SA)	NA
Lee et al,^55^ 2022	Cross-sectional	US/multiple	Adults aged ≥18 y	214 505	8628 (776 SI; 138 SA)	1.36 (1.21-1.53) (SI); 1.40 (1.16-1.70) (SA)	Sex, race and ethnicity, marital status, age, educational level, poverty, employment status, health status and behaviors, mental health challenges
Ha et al,^56^ 2023	Cross-sectional	South Korea/Korean	Adults aged ≥40 y with major STED	2 876 667	1 007 321 (4514 S)	1.33 (1.26-1.41) (S)	Age group, sex, region, socioeconomic status, physical comorbidity, psychiatric hospitalization prior to diagnosis of any major STED
Sung et al,^57^ 2023	Cross-sectional	Taiwan/Taiwanese	All age groups	1 949 101	271 (1 S)	2.00 (1.36-2.66) (S)	Sex, age group, geographical area of residence, urbanization level of residence area, monthly income

Abbreviations: ED, emergency department; EU, European Union; NA, not available; OR, odds ratio; S, suicide; SA, suicide attempt; SI, suicidal ideation; STED, sight-threatening eye disease.

**Table 2.** Definition and Assessment Methods of Visual Impairment and Suicidality

Source	Visual impairment		Definition of suicidal ideation	Definition of suicide attempt	Definition of suicide
	Definition	Assessment method			
Jorm et al,^27^ 1995	Somewhat impaired: could not see photographs or “Interviewer had to read aloud or omit questions requiring sight”	Rating by interviewer	In the last 2 weeks, have you felt as if you wanted to die?	NA	NA
Forsell et al,^28^ 1997	Visual problem grading by physician, only those individuals who had an obvious practical problem causing disability	Examination	Score of ≥2 in Comprehensive Psychopathological Rating Scale	NA	NA
Waern et al,^29^ 2002	NA	Review of medical record and interview	NA	NA	Necropsy at the Gothenburg Institute of Forensic Medicine
Waern et al,^30^ 2003	NA	Interview	NA	NA	Forensic reports of those who were autopsied at the Göteborg Institute of Forensic Medicine
Yip et al,^31^ 2003	2 Items on vision problem	Interview	≥1 of 6 Items of the Geriatric Mental State Examination–Version A	NA	NA
Mojon-Azzi et al,^33^ 2008	General eyesight was classified at 6 levels: 1 indicates excellent; 2, very good; 3, good; 4, fair; 5, poor; and 6, blind	Survey	Measured by mention of suicidal feelings in interview	NA	NA
Lam et al,^32^ 2008	1 Indicates blind in both eyes; 2, VI in both eyes; 3, blind in 1 eye and VI in 1; 4, blind or VI in 1 eye only and 1 with good vision or not mentioned	Survey	NA	NA	Linking the National Health Interview Survey data files and the National Death Index
Fässberg et al,^45^ 2013	Blindness or a defect that made some of the examination tasks impossible to perform despite glasses or the use of a magnifying glass	Examination	1. Have you ever felt that life was not worth living? 2. Have you ever wished you were dead, for instance, that you could go to sleep and not wake up? 3. Have you ever thought of taking your life, even if you would not really do it? 4. Have you ever reached the point where you seriously considered taking your life, or perhaps made plans how you would go about doing it? (5) Have you ever attempted to take your life?	NA	NA
Okamura et al,^41^ 2014	Positive answer to a question (do you have any trouble seeing?)	Survey	In the past 2 wk, have you repetitively wished you were dead? In the past 2 wk, have you repetitively thought of suicide?	NA	NA
Cho et al,^42^ 2015	BCVA of worse than logMAR 0.5 (Snellen, 20/63) in the better-seeing eye	Examination (logMAR)	Have you thought seriously about suicide in the past 12 mo?	NA	NA
Kim et al,^39^ 2015	*ICD-10* VI definition (maximum corrected vision <0.33 in 1 or both eyes)	Examination (logMAR)	Did you ever think that you wanted to die during the past year?	NA	NA
Rim et al,^40^ 2015	Uncorrected VA and/or BCVA at 4 m using an international standard vision chart based on decimal fractions (Jin’s vision chart, Seoul, Korea)^58^	Examination (uncorrected VA and/or BCVA)	Have you ever had suicidal ideation within the past 1 y?	Have you ever attempted suicide within the past 1 y?	NA
Meyer-Rochow et al,^44^ 2015	VA ≥0.05, but <0.3 was defined as low vision, and VA <0.05 was considered blind	Examination	NA	NA	By a forensic examiner
Jung and Park,^43^ 2016	Glaucoma diagnosis based on International Society of Geographical and Epidemiological Ophthalmology criteria	Examination (IOP, VF, optic disc)	During the past year, have you ever had a suicidal ideation?	NA	NA
Morton,^37^ 2017	Participants were asked whether or not they had VI and were provided with 3 response choices (no, 1 eye, both eyes)	Interview	NA	NA	Cause of Death Register
Moses,^34^ 2018	Positive answer to questions: Do you have a disability that limits you from doing certain activity; if yes, which disability?	Survey	NA	During the past 12 mo, have you attempted to kill yourself?	NA
Alvarado-Esquivel,^36^ 2018	NA	Survey	According to definitions of the CDC	According to definitions of the CDC	NA
Cosh et al,^38^ 2019	Parinaud scale ≥3	Examination (near VA, Parinaud scale)	Suicidality item of the Major Depressive Disorder module of the Mini-International Neuropsychiatric Interview	NA	NA
Na et al,^46^ 2019	*ICD-10* codes H53-54	National Health Insurance database	NA	NA	Death Statistics Database based on data of the Korean Statistical Information Service (*ICD-10* codes X60-X84)
Akram and Batool,^35^ 2020	NA	Survey	Urdu form of Suicidal Behavior Questionnaire Revised^59^	Urdu form of Suicidal Behavior Questionnaire Revised^59^	NA
Park and Lee,^47^ 2020	Positive answer to a question (Are you blind or do you have serious difficulty seeing, even when wearing glasses?)	Survey	At any time in the past 12 mo, did you seriously think about trying to kill yourself?	NA	NA
Smith et al,^48^ 2020	VFQ scores range from 0-100, with higher scores indicating more visual ability; VA in logMAR, with higher scores indicating worse acuity and scores of 0.5 or higher categorized as low vision	Examination (logMAR, VF, VFQ)	Modified Beck Scale for Suicide Ideation	NA	NA
Khurana et al,^50^ 2021	Positive answer to questions: “With your glasses (or contact lenses if you wear any), do you have any difficulty seeing ordinary newsprint at arm’s length?” and “With your glasses or contact lenses if you wear any, do you have any difficulty clearly seeing the face of someone across a room, that is, from 4 m or 12 feet away?”	Survey	Have you ever thought of taking your life, even if you would not actually do it?	Have you ever made an attempt to take your life, by taking an overdose of tablets or in some other way?	NA
Okoro et al,^51^ 2021	I am blind or have serious difficulty seeing, even when wearing glasses	Survey	At any time in the past 30 d, did you seriously think about trying to kill yourself?	NA	NA
Parker et al,^52^ 2021	Asked students to report “yes” or “no” as to whether they were blind or had serious difficulty seeing, even when wearing glasses	Interview	Suicidality module of the Mini-International Neuropsychiatric Interview	NA	NA
Marlow et al,^53^ 2021	Are you blind or do you have serious difficulty seeing, even when wearing glasses?	Interview, National Survey on Drug Use and Health	At any time in the past 12 mo, did you seriously think about trying to kill yourself?	During the past 12 mo, did you try to kill yourself?	NA
Marlow et al,^54^ 2022	Are you blind or do you have serious difficulty seeing, even when wearing glasses?	Interview, National Survey on Drug Use and Health	At any time in the past 12 mo, did you seriously think about trying to kill yourself?	During the past 12 mo, did you try to kill yourself?	NA
Smith et al,^49^ 2022	VA worse than 6/18 (0.48 logMAR) in the better-seeing eye	Examination (logMAR)	Did you think of death, or wish you were dead?	During this period, did you ever try to end your life?	NA
Lee et al,^55^ 2022	Asking the respondents whether they were blind or if they had serious difficulty seeing	Survey	At any time in the past 13 mo, did you seriously think about trying to kill yourself?	During the past 13 mo, did you try to kill yourself?	NA
Ha et al,^56^ 2023	BCVA ≤6/100 in better eye; VF ≤5° from visual axis for both eyes	National Health Institute health checkup records and the National Disability Registration for coexisting severe VI	NA	NA	Death by suicide, defined as *ICD-10* codes X60-X84 as recorded in the Korea National Statistical Office
Sung et al,^57^ 2023	*ICD-9* codes 369.3 and 369.4	Diagnostic code from Taiwanese Longitudinal Health Insurance Database	NA	NA	Suicide mortality, defined as *ICD-9* codes E950- E959

Abbreviations: BCVA, best-corrected visual acuity; CDC, Centers for Disease Control and Prevention; *ICD-9*, *International Classification of Diseases, Ninth Revision*; *ICD-10*, *International Statistical Classification of Diseases, Tenth Revision*; IOP, intraocular pressure; logMAR, logarithm of the minimum angle of resolution; NA, not available; VA, visual acuity; VF, visual field; VFQ, visual function questionnaire; VI, visual impairment.

### Visual Impairment and Risk of Suicide

**Figure 2.**
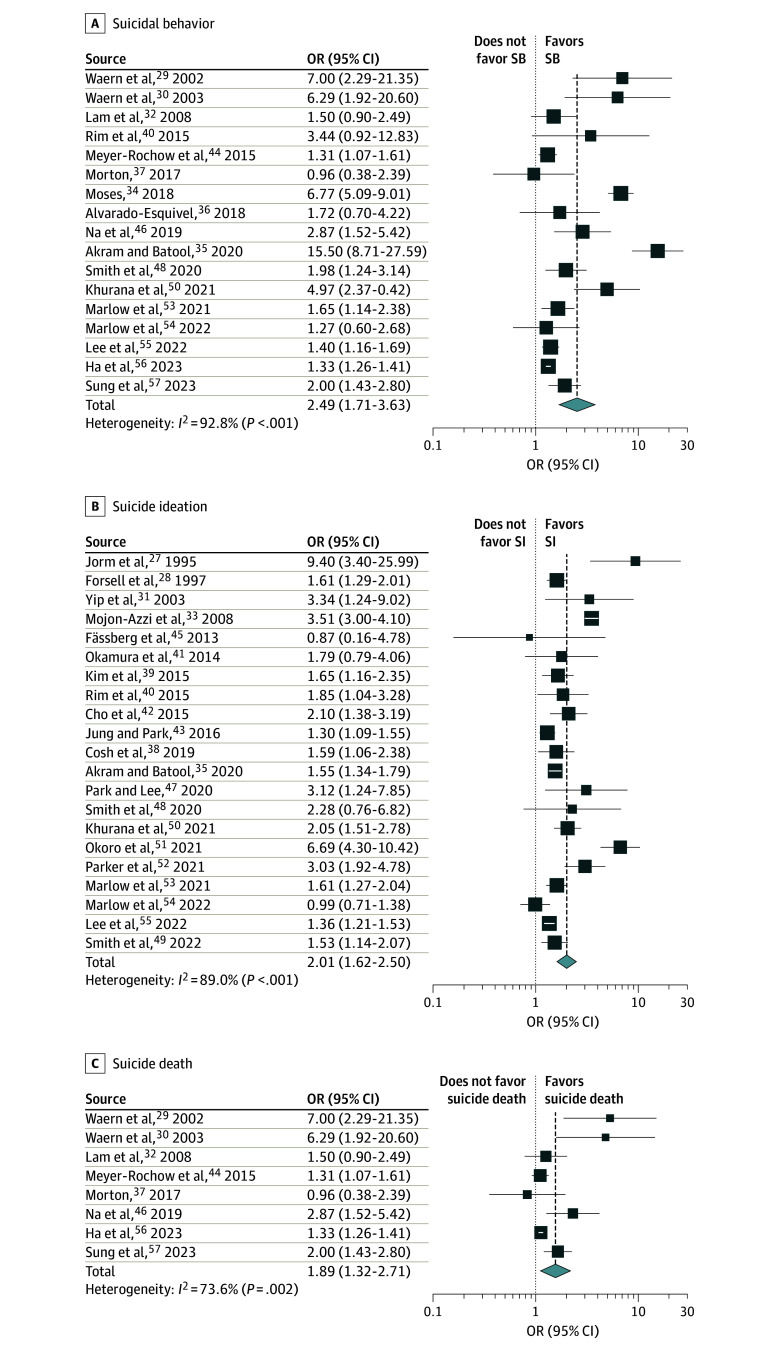
Risk Estimates of Association Between Visual Impairment and Suicidal Tendencies The size of the box representing the point estimate for each study in the forest plot is proportional to the contribution of that study’s weight estimate to the summary estimate. Error bars indicate the 95% CIs. The diamond represents the pooled odds ratio (OR); the lateral tips of the diamond represent the associated 95% CIs. SB indicates suicidal behavior; SI suicidal ideation.

The association between visual impairment and suicidal behavior was investigated in 17 studies^29,30,32,34,35,36,37,40,44,46,48,50,53,54,55,56,57^ with a total of 5 602 285 participants. Our summary estimate of visual impairment as a risk factor for suicidal behavior was an OR of 2.49 (95% CI, 1.71-3.63), with heterogeneity of *I^2^* = 92.8% (*P* < .001) (Figure 2A). A total of 21 studies^27,28,31,33,35,38,39,40,41,42,43,45,47,48,49,50,51,52,53,54,55^ assessed suicidal ideation, encompassing 611 899 participants. The pooled OR for the association between visual impairment and suicidal ideation was 2.01 (95% CI, 1.62-2.50), with heterogeneity of *I^2^* = 89.0% (*P* < .001) (Figure 2B). Eight studies^29,30,32,37,44,46,56,57^ involving a total of 5 067 113 participants examined the association between visual impairment and the risk of suicide death. The pooled OR was 1.89 (95% CI, 1.32-2.71), with heterogeneity of *I^2^* = 73.6% (*P* = .002) (Figure 2C). Drapery plots in eFigures 1 and 2 in Supplement 1 show the different meta-analytic results by *P* value functions.

### Moderators of Suicide Risk

Meta-regression analyses were undertaken to establish the association between individual moderators (predictive factors) and the pooled effect size (ie, risk of suicidal behavior). Within the group of 8 moderators, the mean age of study participants was a notable risk factor for suicidal behavior. Specifically, 71.3% of the variance in true effect sizes could be attributed to age. The Test of Moderators also showed significance (*P* < .001) (eTable 2 in Supplement 1). This signifies that the predictive factor—namely, the mean age of study participants—indeed was associated with the effect sizes of the studies.

### Subgroup Analysis on Study Participants’ Mean Age

Random-effects meta-regression analyses showed that participant age was a possible risk factor. Thus, we performed a subgroup analysis comparing effect sizes according to mean age (eTable 3 in Supplement 1). The pooled OR for studies that had included adolescent patients was 9.85 (95% CI, 4.39-22.10), representing the highest value among the various age groups. The second-highest OR was observed among individuals older than 65 years at 6.66 (95% CI, 2.95-15.00).

### Combined Factors Contributing to Suicide Risk

Multiple meta-regression analyses were performed to identify the blend of multiple moderators predictive of the pooled effect size, while also considering interactions among moderators. The most optimal model for estimating risk of suicidal behavior in patients with visual impairment was the blend of moderators (Akaike information criterion corrected, 29.7), encompassing mean age of study participants (model importance, 0.99), consideration of potential confounding factors in a study (model importance, 0.27), and country where the study was conducted (model importance, 0.21). A model-averaged plot of predictive factor importance displays the significance of each factor across all of the models (eFigure 3 in Supplement 1). The results for predictive factors associated with suicidal ideation risk in patients with visual impairment are plotted in eFigure 4 in Supplement 1.

### GOSH Plot and Sensitivity Analysis

Our results showed 2 peaks suggestive of the effect size heterogeneity patterns for the association between visual impairment and risk of suicidal behavior (eFigure 5 in Supplement 1). The 3 clustering algorithms for detection of different clusters in a GOSH plot were applied and detected 2 studies^34,35^ that might have contributed to cluster imbalance. After excluding these 2 potential outliers, we again performed the meta-analysis and obtained 1.83 (95% CI, 1.48-2.28) as the pooled OR for visual impairment and risk of suicidal behavior (eTable 4 in Supplement 1).

### Publication Bias

**Figure 3.**
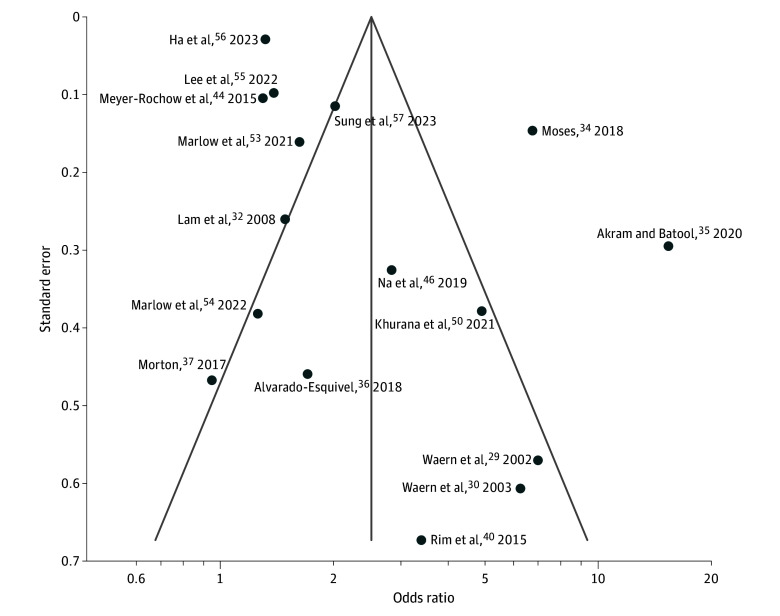
Publication Bias Regarding Visual Impairment and Risk of Suicidal Behavior The middle line indicates the overall effect of the meta-analysis, while the 2 lines on either side represent the 95% CIs.

Figure 3 shows a funnel plot illustrating the potential publication bias. The studies are distributed around the pooled effect size (indicated by the vertical line at the center), both within and outside the funnel’s contours. This suggests a reduced likelihood of significant bias in smaller studies, which are more susceptible to yielding nonsignificant results and thus potentially going unnoticed. Funnel plots for suicidal ideation and suicide death are shown in eFigure 6 in Supplement 1.

## Discussion

Our comprehensive meta-analysis of 31 population-based studies revealed an association between visual impairment and elevated risk of suicide encompassing suicidal behavior, suicidal ideation, and suicide death. Notably, through multiple meta-regression analyses, we uncovered a particularly pronounced risk of suicide associated with visual impairment among adolescents.

For individuals with visual impairment, the underlying causes of suicidal behavior may be complex and multifactorial. A nationwide survey conducted in the US found that approximately 88% of respondents regarded eye health as a critical component of their overall well-being, with blindness being ranked as the most severe conceivable health outcome.^60^ Indeed, visual impairment has implications that extend beyond the confines of clinical ophthalmology. Systematic examination of and consultation with patients with visual impairment consistently reveal a concerning level of compromised quality of life, reduced physical activity, social isolation, decline in autonomy, diminished personal income, and substantial prevalence of depression.^9,61,62^ Notably, these factors are widely recognized as significant risk factors for suicide.^63^

In the literature, 2 meta-analyses have investigated the potential association between visual impairment and suicide. Rajeshkannan et al^64^ identified a correlation between suicidal ideation (OR, 1.53 [95% CI, 1.30-1.79]) or suicide attempt (OR, 4.55 [95% CI, 2.39-8.67]) and visual impairment based on 6 relevant studies. Palbo et al,^65^ having integrated 8 studies into a quantitative analysis, also posited elevated risks of suicide death (OR, 7.00 [95% CI, 2.30-21.40]), suicide attempt (OR, 2.62 [95% CI, 1.29-5.31]), and suicidal ideation (OR, 1.83 [95% CI, 1.40-2.40]) among individuals with visual impairment. However, the restricted number of studies integrated into their analysis impeded Palbo et al^65^ from achieving precise measurements of risk magnitude and constrained their potential to conduct additional analyses. Our report encompasses 31 studies identified through meticulous literature searches, thus allowing for comprehensive summary estimates regarding the association between visual impairment and risk of suicide and facilitating the execution of meta-regression analyses.

In our results, studies focusing on adolescents with visual impairment demonstrated the highest risk of suicidal behavior. Adolescence is a complex stage of life in which both physiological and psychological changes begin. In this period, individuals cultivate independence and build social networks by acquiring new skills and knowledge and navigating educational and interpersonal ups and downs.^66^ In the studies scrutinized, symptoms related to anxiety, tension, and general distress were significantly higher in adolescents with visual impairment than in those without.^67^ Through interviews with adolescents with visual impairment, Rainey et al^68^ demonstrated that these individuals had significant concerns about their future lives. They voiced worries about facing potential prejudice from future employers, managing independent living in unfamiliar surroundings, and shouldering the sole responsibility for household matters, including finances.

### Limitations

This study has some limitations. First, heterogeneities between the examined studies warrant attention. The study population differed to a certain degree; for example, some studies focused on elderly individuals, 1 included homeless individuals, 2 focused on patients with vision-related diagnoses, and others had a broader demographic focus. Although we performed a rigorous sensitivity analysis to validate the results, a potential association between study heterogeneities and the pooled effect remains. Second, methods used to assess the outcome varied among the studies. Parameters related to suicide were evaluated using questions in different phraseology, language, and time frames of interest. Third, variations in the definition, classification, and assessment methods for visual impairment in each study could potentially confound the association between visual impairment and suicide risk. Specifically, objective assessment of vision might better reflect associated ocular diseases and/or physiological decline. Conversely, self-reported vision may be a marker of functional performance in activities of daily living and may be exacerbated by psychological distress such as depression and social isolation. Our model-averaged plots of predictive factor importance, which illustrate the significance of each factor across all models, revealed that the vision assessment methods did not emerge as a significant factor. This suggests that the association between visual impairment and suicide risk might be independent of the types of vision assessment methods used in the studies. However, we believe that it is crucial to acknowledge the discrepancies in definitions and assessments of the degree of visual impairment across studies as significant factors in interpreting the results. Fourth, although most of the studies included in the analysis adjusted for depression and other important risk factors, the potential confounding of additional risk factors cannot be ruled out. Since the etiology of suicidal behavior is complex, and because diverse risk factors are associated with different individual and cultural contexts, further research is warranted to determine which factors may modulate the risk of suicide in patients with visual impairment.

## Conclusions

In this systematic review and meta-analysis of visual impairment and suicide risk, an association between visual impairment and an increased risk of suicide was identified. This finding emphasizes the importance of eye health to overall mental well-being. It is recommended that clinicians remain attentive to the elevated risk and be ready to implement suitable suicide prevention measures when required, especially when dealing with adolescents. In addition, the limited number of studies addressing adolescents with visual impairment and suicide highlights the importance of conducting additional research in this area.

## Notes

Supplement 1. **eAppendix 1.** Search Terms **eAppendix 2.** Quality Assessment According to the Newcastle-Ottawa Scale **eFigure 1.** Drapery Plot—Suicidal Behavior **eFigure 2.** Drapery Plot—Suicidal Ideation and Death by Suicide **eFigure 3.** Model-Averaged Plot of Predictive Factor Importance—Suicidal Behavior **eFigure 4.** Model-Averaged Plot of Predictive Factor Importance—Suicidal Ideation **eFigure 5.** Graphic Display of Heterogeneity (GOSH) Plot **eFigure 6.** Funnel Plots for Suicidal Ideation and Suicide Death **eTable 1.** Covariates for Meta-Regression **eTable 2.** Effect Moderators for Studies on Suicidal Behavior **eTable 3.** Subgroup Analysis on Study Participants’ Mean Age **eTable 4.** Association Between Visual Impairment and Suicidality Risk After Exclusion of Potential Outliers
eReferences

Supplement 2.
Data Sharing Statement
