# Error in Author Affiliations

**DOI:** 10.1001/jamanetworkopen.2024.18308

**Published:** 2024-05-21

In the Original Investigation titled “Visual Impairment and Suicide Risk: A Systematic Review and Meta-Analysis,”^1^ published April 17, 2024, the author affiliation given for Sung Ryul Shim was incorrect; Dr Shim’s affiliation during the conduct of the study was Department of Biomedical Informatics, College of Medicine, Konyang University, Daejeon, Korea. This article has been corrected.
