# Supplementary material for: Visual Impairment and Suicide Risk: A Systematic Review and Meta-Analysis
Source: JAMA Netw Open. 2024 Apr 17;7(4):e247026. doi: 10.1001/jamanetworkopen.2024.7026 (PMC11024775; doi:10.1001/jamanetworkopen.2024.7026)
Supplement: Supplement 1. — eAppendix 1. Search Terms eAppendix 2. Quality Assessment According to the Newcastle-Ottawa Scale eFigure 1. Drapery Plot—Suicidal Behavior eFigure 2. Drapery Plot—Suicidal Ideation and Death by Suicide eFigure 3. Model-Averaged Plot of Predictive Factor Importance—Suicidal Behavior eFigure 4. Model-Averaged Plot of Predictive Factor Importance—Suicidal Ideation eFigure 5. Graphic Display of Heterogeneity (GOSH) Plot eFigure 6. Funnel Plots for Suicidal Ideation and Suicide Death eTable 1. Covariates for Meta-Regression eTable 2. Effect Moderators for Studies on Suicidal Behavior eTable 3. Subgroup Analysis on Study Participants’ Mean Age eTable 4. Association Between Visual Impairment and Suicidality Risk After Exclusion of Potential Outliers eReferences [file jamanetwopen-e247026-s001.pdf]

# Supplementary Online Content

Kim CY, Ha A, Shim SR, Hong IH, Chang IB, Kim YK. Visual impairment and suicide risk: a systematic review and meta-analysis. *JAMA Netw Open*. 2024;7(4):e247026. doi:10.1001/jamanetworkopen.2024.7026

**eAppendix 1.** Search Terms
**eAppendix 2.** Quality Assessment According to the Newcastle-Ottawa Scale
**eFigure 1.** Drapery Plot—Suicidal Behavior
**eFigure 2.** Drapery Plot—Suicidal Ideation and Death by Suicide
**eFigure 3.** Model-Averaged Plot of Predictive Factor Importance—Suicidal Behavior
**eFigure 4.** Model-Averaged Plot of Predictive Factor Importance—Suicidal Ideation
**eFigure 5.** Graphic Display of Heterogeneity (GOSH) Plot
**eFigure 6.** Funnel Plots for Suicidal Ideation and Suicide Death
**eTable 1.** Covariates for Meta-Regression
**eTable 2.** Effect Moderators for Studies on Suicidal Behavior
**eTable 3.** Subgroup Analysis on Study Participants' Mean Age
**eTable 4.** Association Between Visual Impairment and Suicidality Risk After Exclusion of Potential Outliers
**eReferences**

This supplementary material has been provided by the authors to give readers additional information about their work.

© 2024 Kim CY et al. *JAMA Network Open*.

**eAppendix 1.** Search Terms

**1. Pubmed (MEDLINE)**

(“Vision, low”[Mesh] OR “Low vision”[Tiab] OR “Blindness”[Mesh] OR “Blind*”[Tiab] OR “Visually impaired persons”[Mesh] OR “Sensory”[Tiab] OR “Visual acuity”[Mesh] OR “Visual”[Tiab] OR “Impairment”[Tiab] OR “Disability”[Tiab]) AND (“Suicide”[Mesh] OR “Suicid*”[Tiab] OR “Self-injurious behavior”[Mesh] OR “Self mutilation”[Mesh] OR “Drug overdose”[Mesh])

**2. Cochrane Central Register of Controlled Trials (CENTRAL) in The Cochrane Library (Wiley)**

#1 MeSH descriptor Vision, Low

#2 MeSH descriptor Blindness

#3 MeSH descriptor Visually Impaired Persons

#4 MeSH descriptor Visual Acuity

#5 “Low vision” OR “Blind*” OR “Sensory” OR “Visual” OR “Impairment” OR “Disability”

#6 (#1 OR #2 OR #3 OR #4 OR #5)

#7 MeSH descriptor Suicide

#8 MeSH descriptor Self-injurious behavior

#9 MeSH descriptor Self mutilation

#10 MeSH descriptor Drug overdose

#11 “Suicid*”

#12 (#7 OR #8 OR #9 OR #10 OR #11)

#13 (#6 AND #12)

**3. EMBASE (Ovid)**

(‘Low vision’/exp OR Blindness/exp OR ‘Visually Impaired Persons’/exp OR ‘Visual Acuity’/exp OR Blind* OR “Sensory” OR “Visual” OR “Impairment” OR “Disability”) AND (Suicide/exp OR Automutilation/exp OR ‘Drug overdose’/exp OR Suicid*

© 2024 Kim CY et al. *JAMA Network Open*.

**eAppendix 2.** Quality Assessment According to the Newcastle-Ottawa Scale

| Study | Design | Selection* | Comparability† | Outcome‡ | Total score |
|---|---|---|---|---|---|
| Jorm et al,[1] 1995 | Cross-sectional | 4 | 1 | 3 | 8 |
| Forsell et al,[2] 1997 | Cross-sectional | 3 | 0 | 2 | 5 |
| Waern et al,[3] 2002 | Case-control | 2 | 0 | 3 | 5 |
| Waern et al,[4] 2003 | Cross-sectional | 2 | 0 | 3 | 5 |
| Yip et al,[5] 2003 | Cross-sectional | 4 | 0 | 3 | 7 |
| Mojon-Azzi et al,[6] 2008 | Cross-sectional | 4 | 1 | 3 | 8 |
| Lam et al,[7] 2008 | Cross-sectional | 4 | 1 | 3 | 8 |
| Fässberg et al,[8] 2013 | Cross-sectional | 3 | 0 | 3 | 6 |
| Okamura et al,[9] 2014 | Cross-sectional | 3 | 1 | 2 | 6 |
| Cho et al,[10] 2015 | Cross-sectional | 4 | 0 | 3 | 7 |
| Kim et al,[11] 2015 | Cross-sectional | 4 | 2 | 3 | 9 |
| Rim et al,[12] 2015 | Cross-sectional | 4 | 1 | 3 | 8 |
| Meyer-Rochow et al,[13] 2015 | Cross-sectional | 2 | 0 | 2 | 4 |
| Jung and Park,[14] 2016 | Cross-sectional | 3 | 0 | 2 | 5 |
| Morton,[15] 2017 | Cross-sectional | 3 | 0 | 2 | 5 |
| Moses,[16] 2018 | Cross-sectional | 4 | 0 | 3 | 7 |
| Alvarado-Esquivel,[17] 2018 | Cross-sectional | 2 | 0 | 2 | 4 |
| Cosh et al,[18] 2019 | Cohort | 4 | 0 | 3 | 7 |
| Na et al,[19] 2019 | Cross-sectional | 4 | 2 | 3 | 9 |
| Akram and Batool,[20] 2020 | Cross-sectional | 3 | 0 | 3 | 6 |
| Park and Lee,[21] 2020 | Cross-sectional | 4 | 1 | 3 | 8 |
| Smith et al,[22] 2020 | Cross-sectional | 3 | 0 | 2 | 5 |
| Khurana et al,[23] 2021 | Cross-sectional | 4 | 1 | 3 | 8 |
| Okoro et al,[24] 2021 | Cross-sectional | 4 | 0 | 3 | 7 |

© 2024 Kim CY et al. *JAMA Network Open*.

| Study | Design | Selection* | Comparability† | Outcome‡ | Total score |
|---|---|---|---|---|---|
| Parker et al,[25] 2021 | Cross-sectional | 4 | 0 | 3 | 7 |
| Marlow et al,[26] 2021 | Cross-sectional | 4 | 2 | 3 | 9 |
| Marlow et al,[27] 2022 | Cross-sectional | 4 | 2 | 3 | 9 |
| Smith et al,[28] 2022 | Cross-sectional | 4 | 0 | 3 | 7 |
| Lee et al,[29] 2022 | Cross-sectional | 4 | 2 | 3 | 9 |
| Ha et al,[30] 2023 | Cross-sectional | 4 | 2 | 3 | 9 |
| Sung et al,[31] 2023 | Cross-sectional | 4 | 1 | 3 | 8 |

For each of the sections Selection, Comparability, and Outcome/Exposure, a maximum of 4, 2, and 3 points could be given, respectively. Thus the maximum score was 9. A higher score means higher quality.

*1. Representativeness of the individuals with visual impairment (VI) 2. Definition of VI status 3. Definition of suicide related factor 4. Sample size calculation and achieved adequate sample.

†1. Assesses and adjusts for mental disorders 2. Assesses and adjusts for physical disorders.

‡1. Reliability of assessment of outcome 2. Validated scale or trained health professional to assess outcome 3. Adequate statistical analyses.

© 2024 Kim CY et al. *JAMA Network Open*.

**eFigure 1.** Drapery Plot—Suicidal Behavior

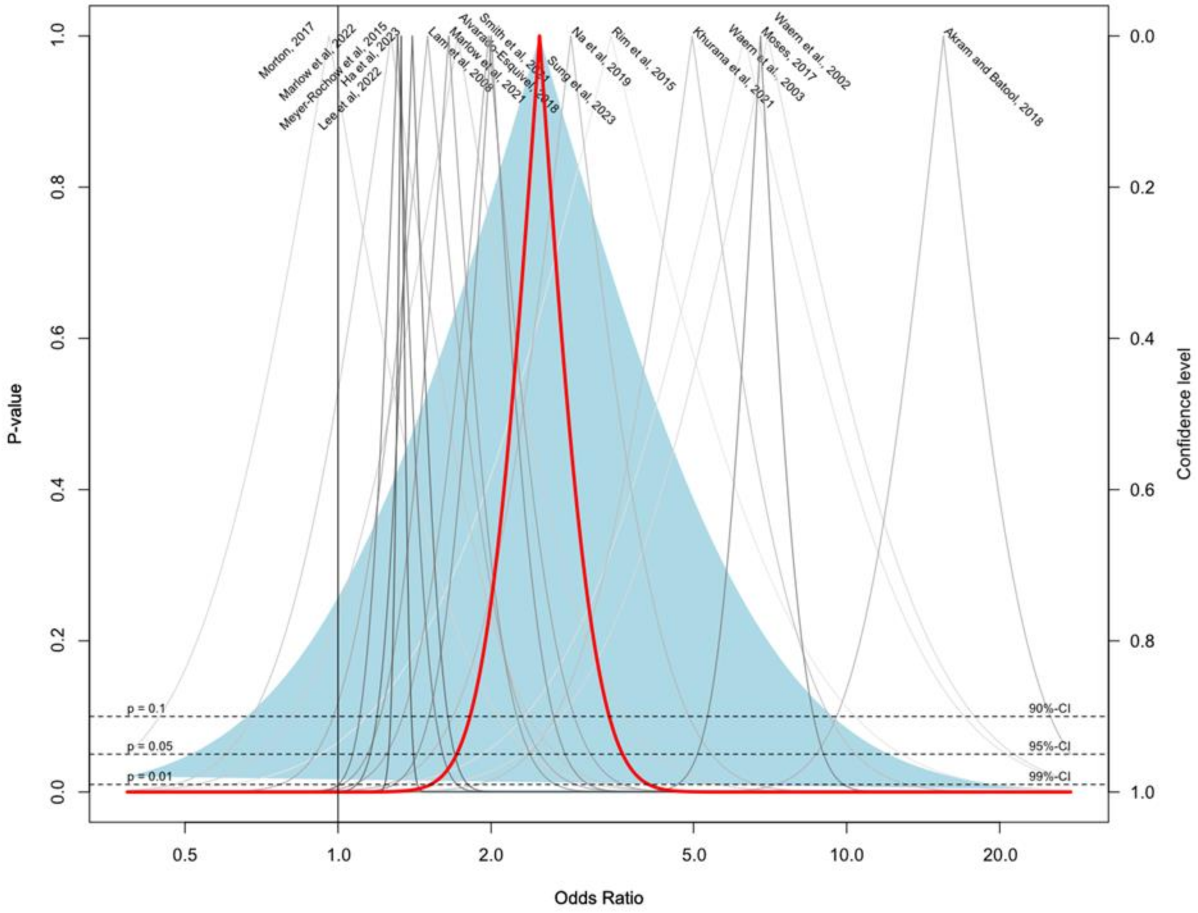


A drapery plot presents the x-axis as the effect size and the y-axis as the *P* value. Gray curves correspond to primary studies, with study weights from the random effects model represented on a grayscale (studies with higher precision shown in dark gray, studies with low precision in light gray). Each point estimate can be read off at the peak of the respective curve. The prediction region (light blue) is broader than the *P* value curve of the pooled estimates (red and black curves), indicating heterogeneity. Horizontal dashed lines can be used to identify confidence intervals for *P* values of 0.1, 0.05, and 0.01.

© 2024 Kim CY et al. *JAMA Network Open*.

**eFigure 2.** Drapery Plots—Suicidal Ideation and Death by Suicide

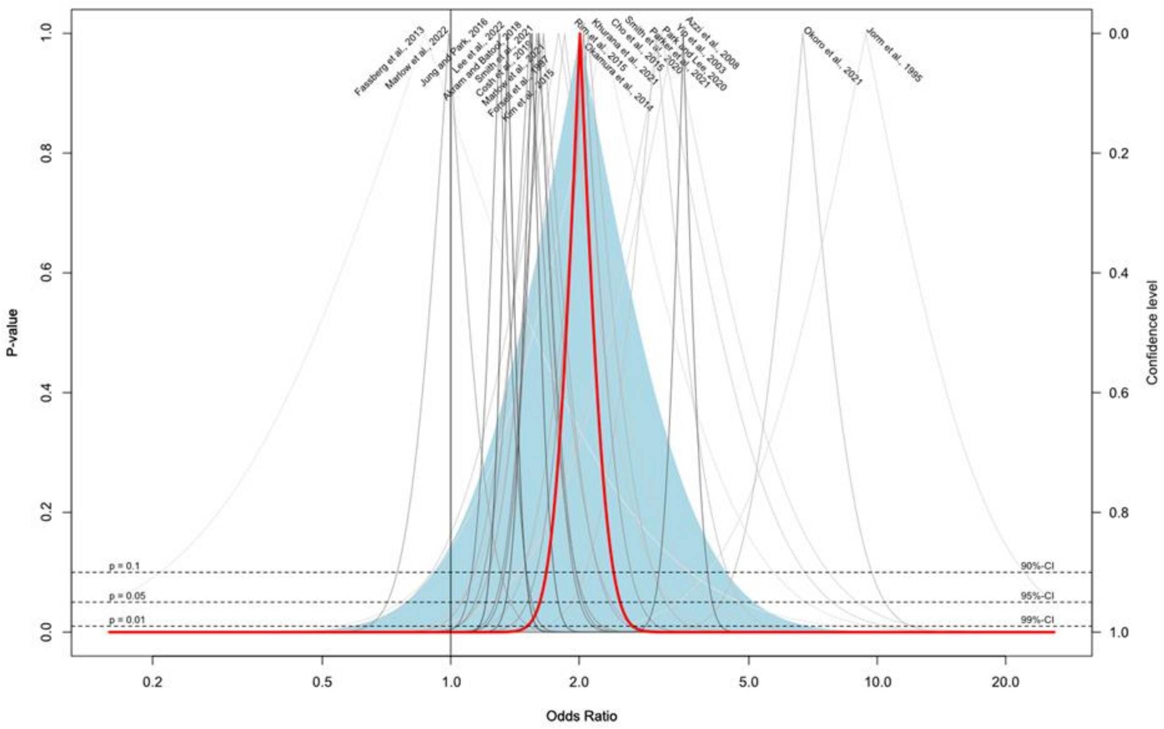


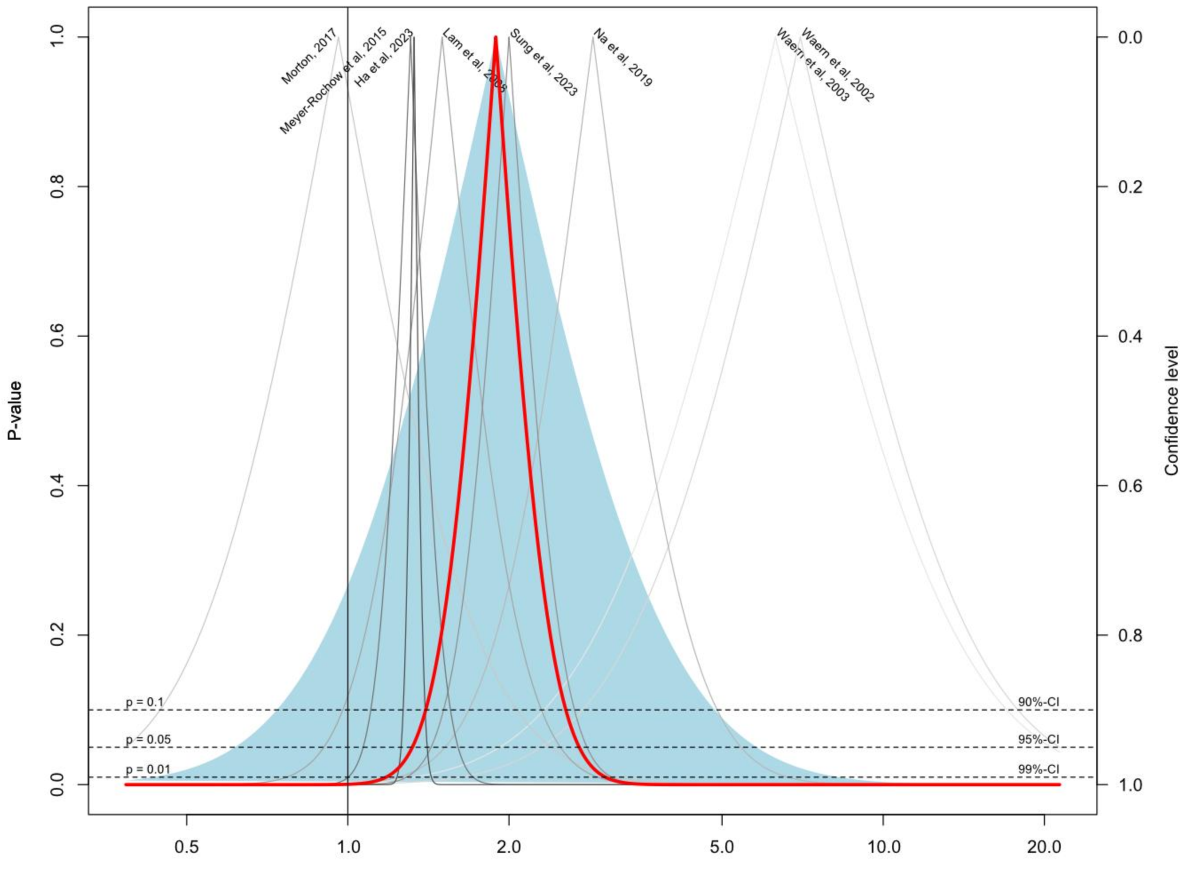


© 2024 Kim CY et al. *JAMA Network Open*.

**eFigure 3.** Model-Averaged Plot of Predictive Factor Importance—Suicidal Behavior

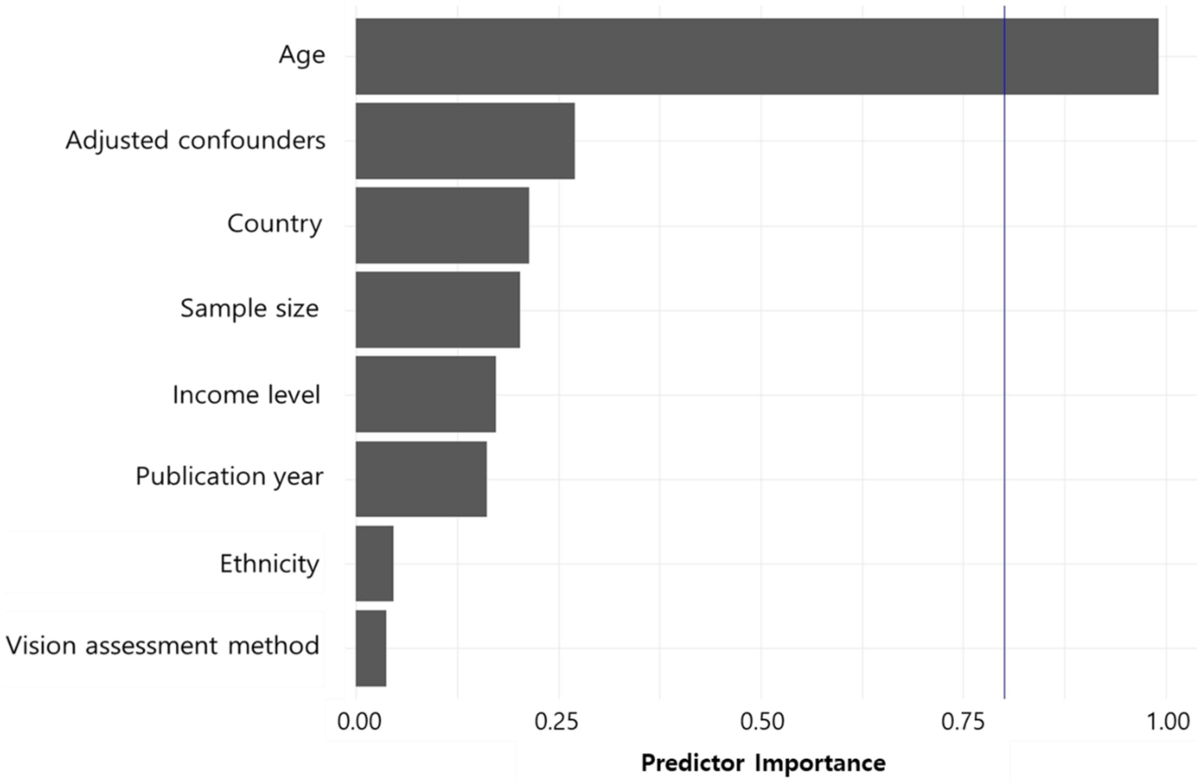


In the model-averaged predictor importance plot, the averaged importance of each predictor across all models is displayed. We can see that the age (predictor importance: 0.99) is the most important predictor.

© 2024 Kim CY et al. *JAMA Network Open*.

**eFigure 4.** Model-Averaged Plot of Predictive Factor Importance—Suicidal Ideation

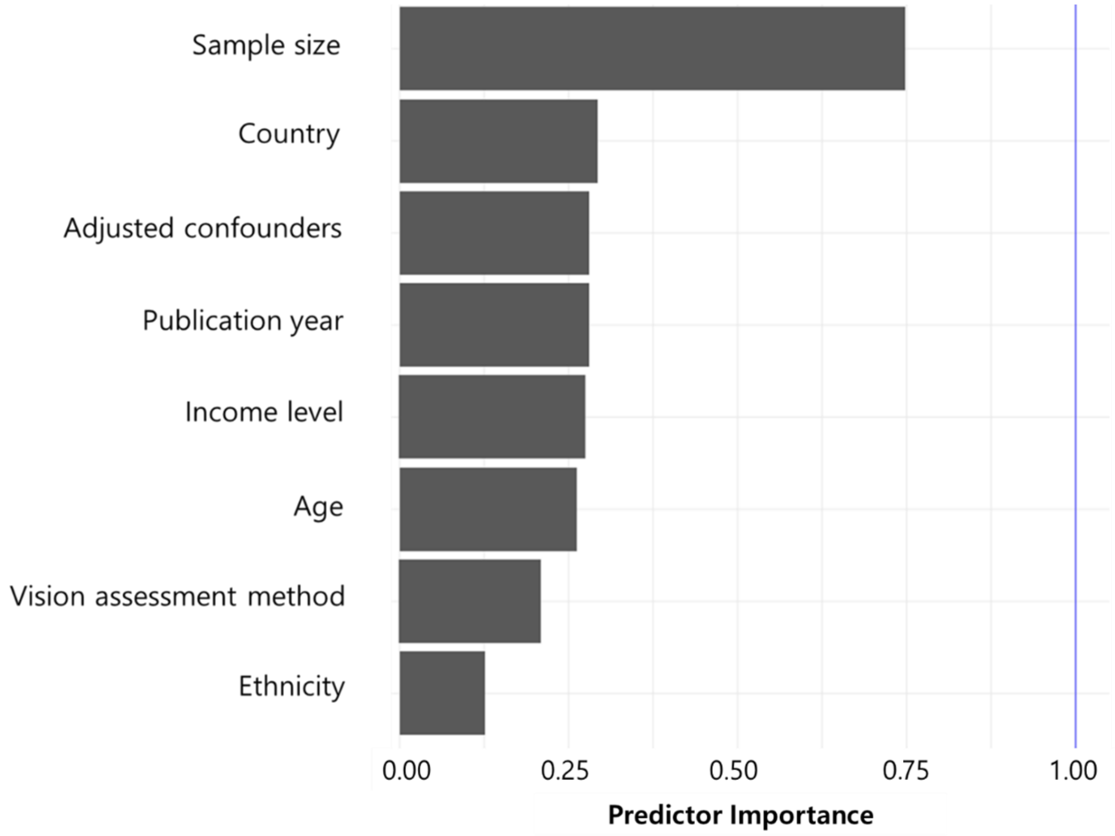


In the model-averaged predictor importance plot, the averaged importance of each predictor across all models is displayed. All predictors did not reach the level of significant importance.

© 2024 Kim CY et al. *JAMA Network Open*.

**eFigure 5.** Graphic Display of Heterogeneity (GOSH) Plot

(A) GOSH plot: effect-size-heterogeneity patterns in data showing two subclusters.

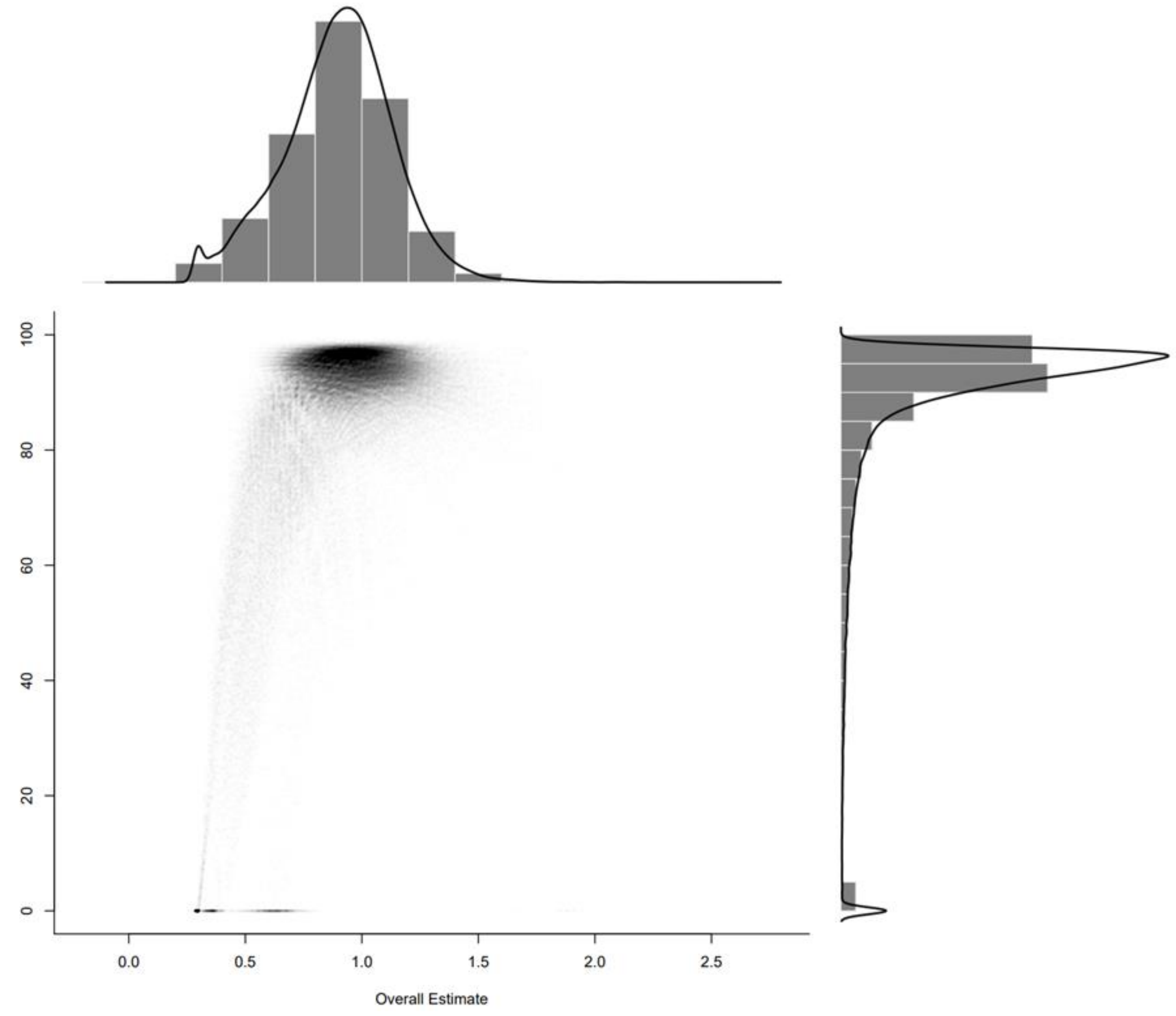


© 2024 Kim CY et al. *JAMA Network Open*.

(B) k-means clustering algorithm

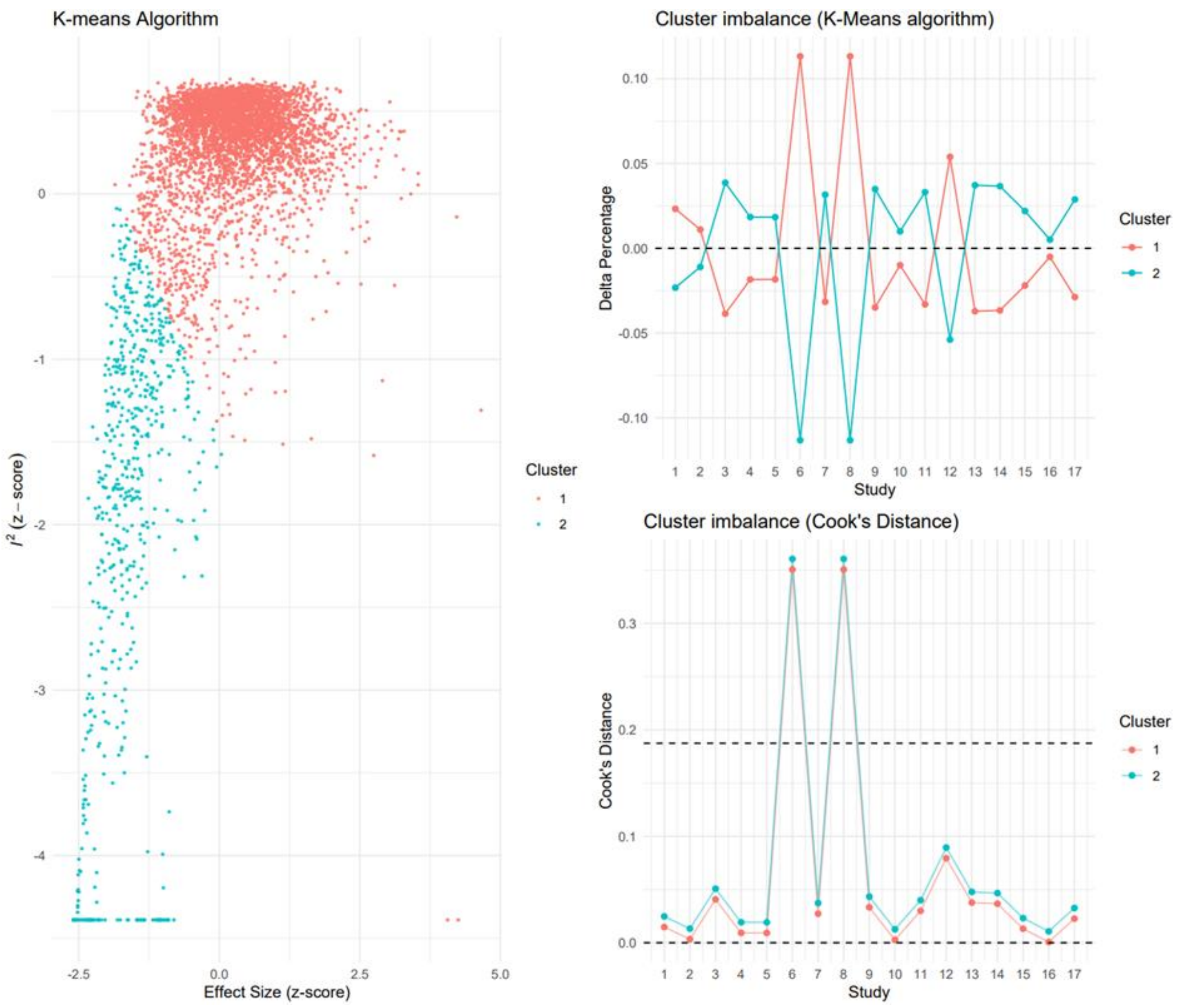


© 2024 Kim CY et al. *JAMA Network Open*.

(C) density-based spatial clustering of applications with noise [DBSCAN] algorithm

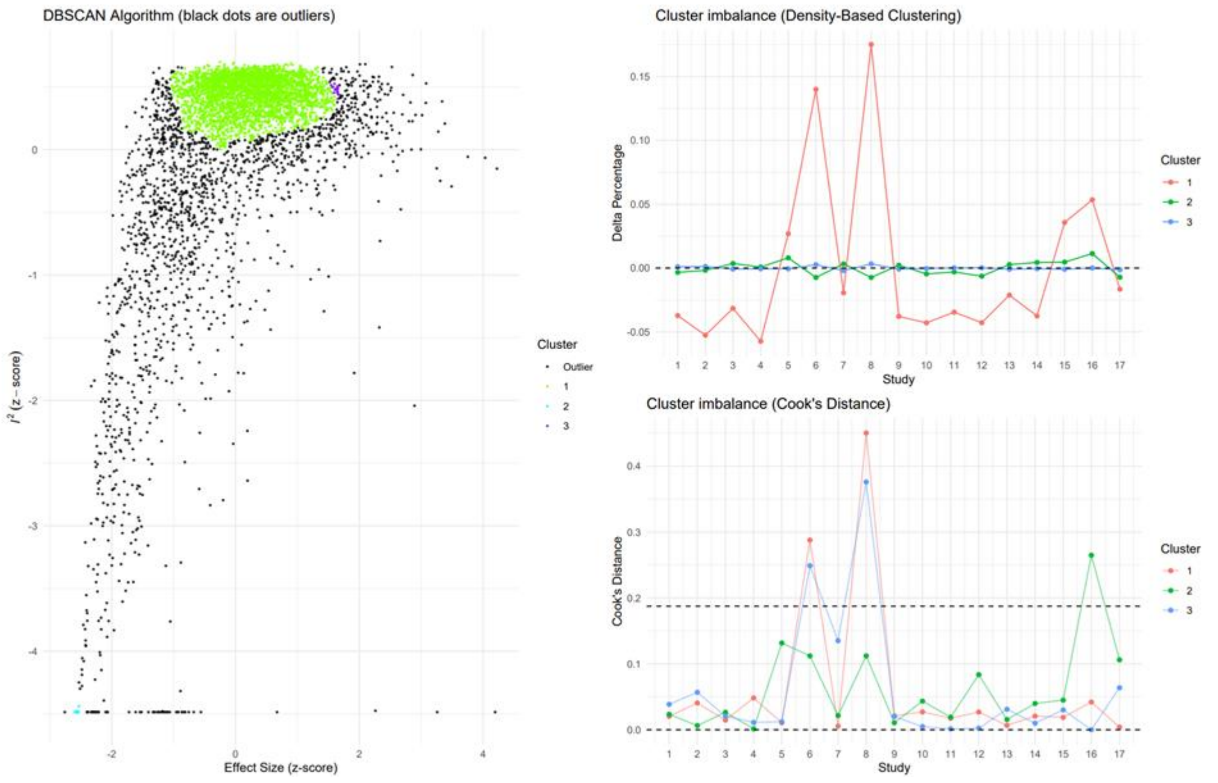


(D) Gaussian mixture model

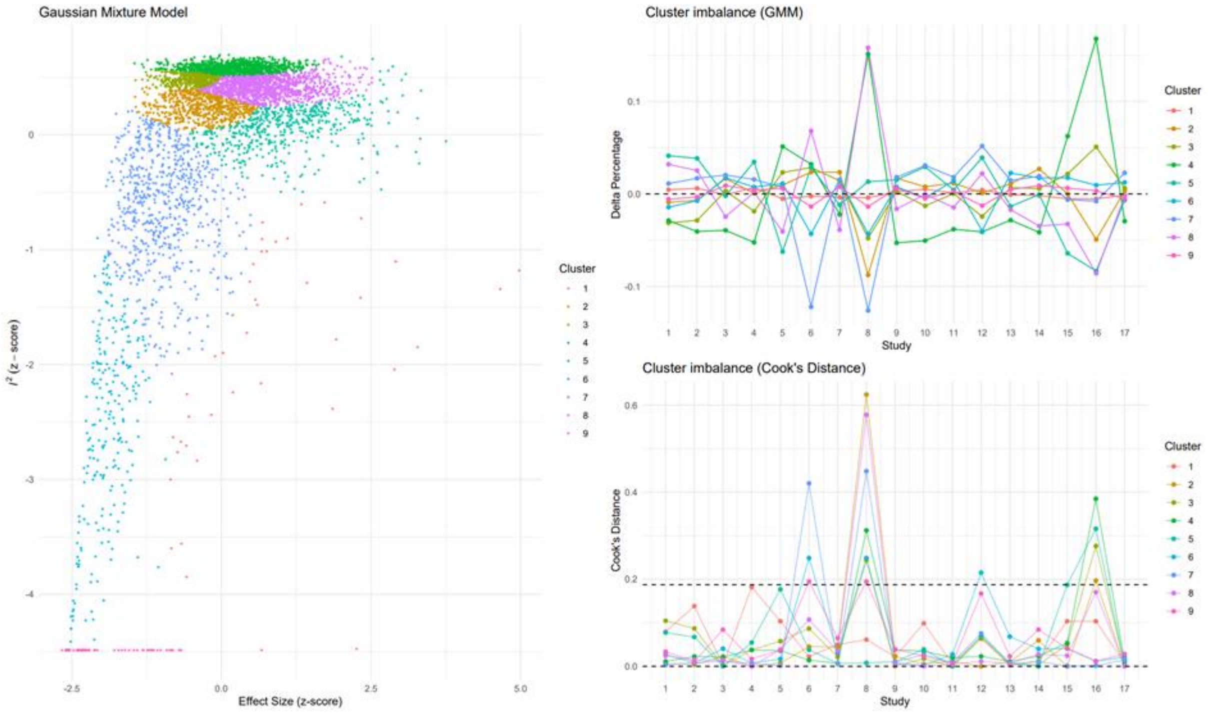


© 2024 Kim CY et al. *JAMA Network Open*.

| **Study number** | |
|---|---|
| 1 | Moses,[16] 2018 |
| 2 | Akram and Batool,[20] 2020 |
| 3 | Alvarado-Esquivel,[17] 2018 |
| 4 | Morton,[15] 2017 |
| 5 | Rim et al,[12] 2015 |
| 6 | Lam et al,[7] 2008 |
| 7 | Waern et al,[3] 2002 |
| 8 | Waern et al,[4] 2003 |
| 9 | Smith et al,[28] 2022 |
| 10 | Khurana et al,[23] 2021 |
| 11 | Meyer-Rochow et al,[13] 2015 |
| 12 | Na et al,[19] 2019 |
| 13 | Marlow et al,[26] 2021 |
| 14 | Marlow et al,[27] 2022 |
| 15 | Lee et al,[29] 2022 |
| 16 | Ha et al,[30] 2023 |
| 17 | Sung et al,[31] 2023 |

© 2024 Kim CY et al. *JAMA Network Open*.

**eFigure 6.** Funnel Plots for Suicidal Ideation and Suicide Death

(A) Suicidal ideation

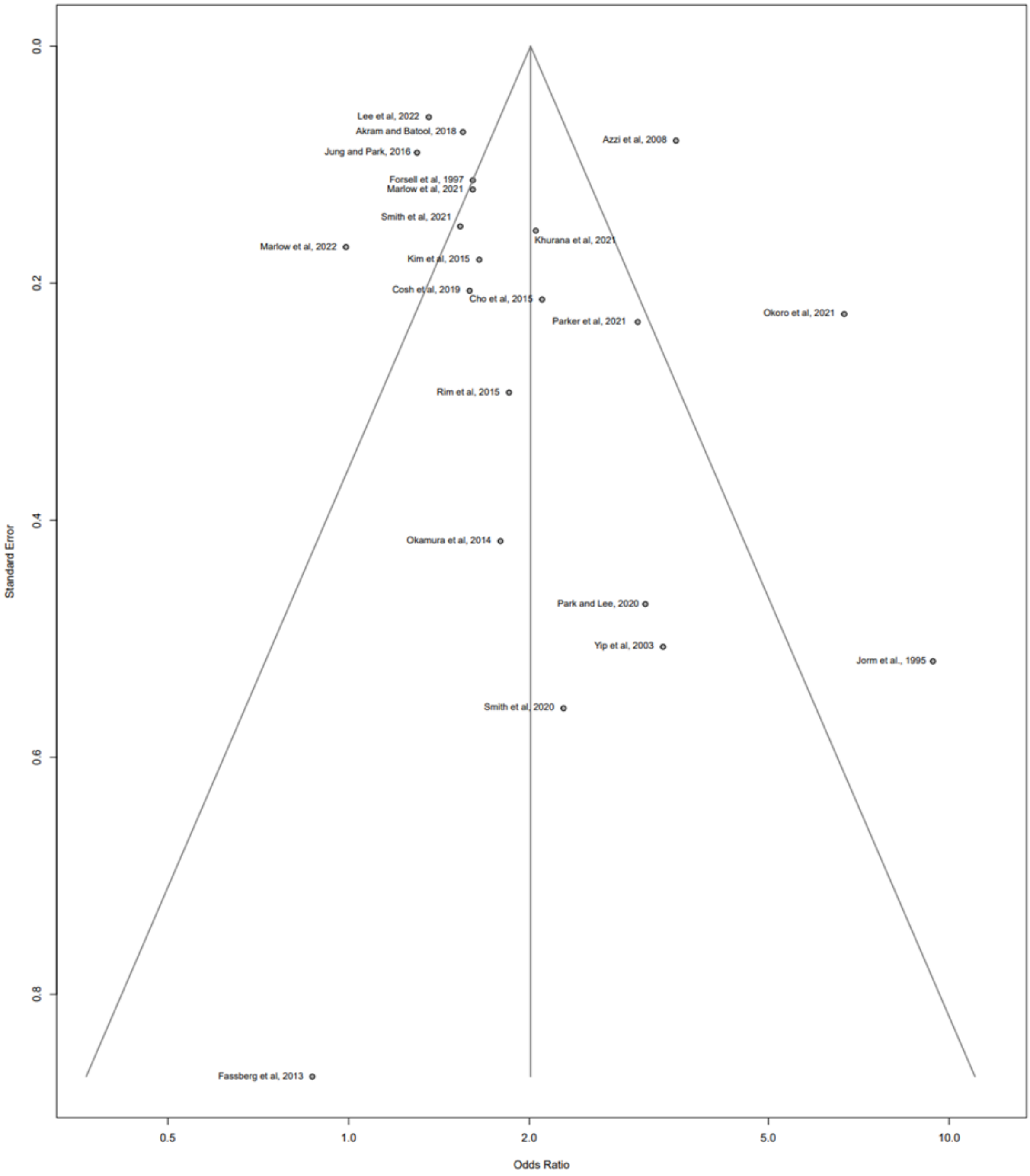


© 2024 Kim CY et al. *JAMA Network Open*.

(B) Death by suicide

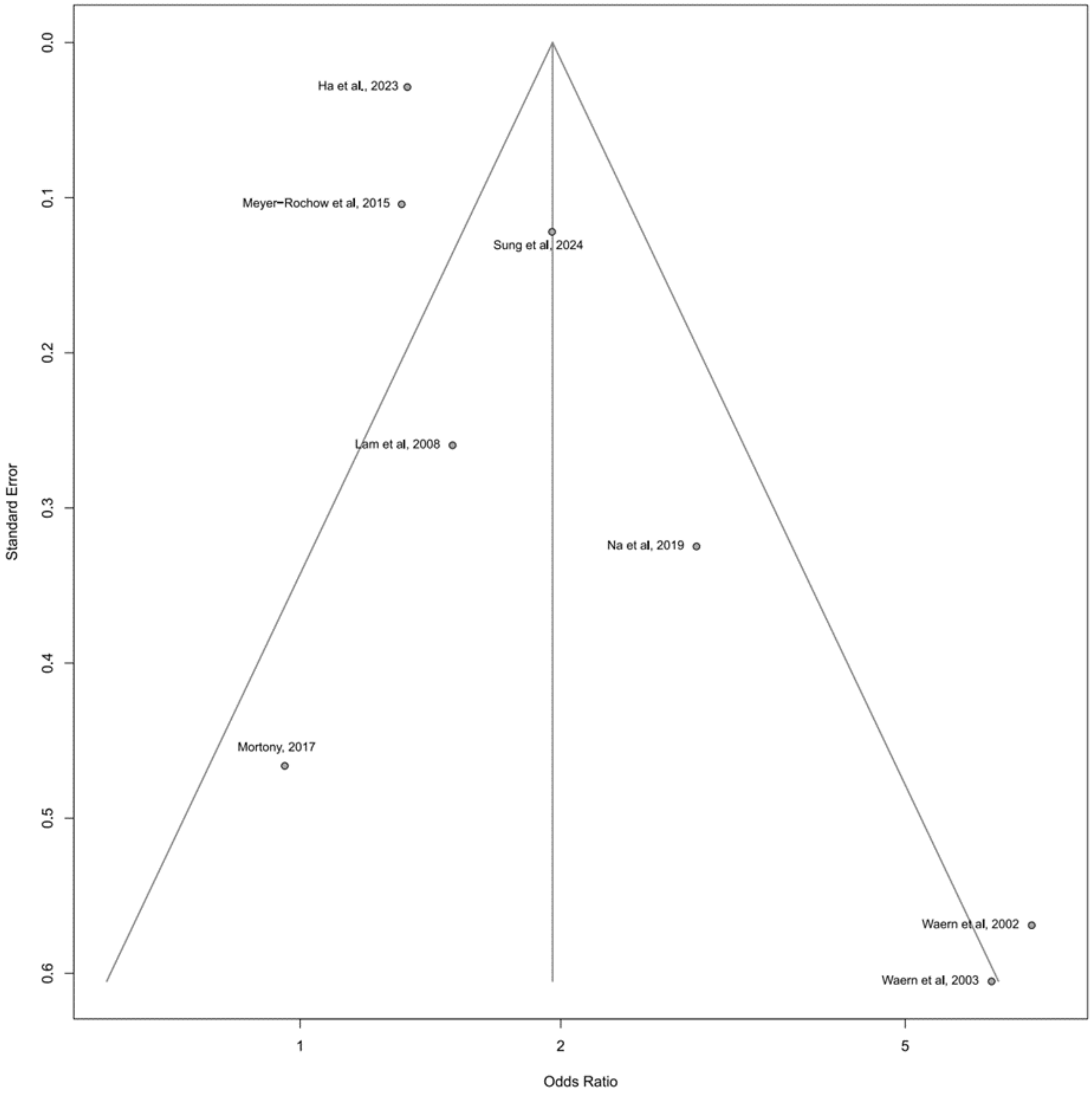


© 2024 Kim CY et al. *JAMA Network Open*.

**eTable 1.** Covariates for Meta-Regression

| Variables | Definitions |
|---|---|
| **Publication year** | Before 2009 |
| | 2010s |
| | 2020s |
| **Ethnicity** | White |
| | Asian |
| | Mix |
| **Age** | All adults |
| | ≥ 40 years |
| | ≥ 50 years |
| | ≥ 65 years |
| | Adolescents |
| **Sample size** | Smaller than 1000 |
| | 1,000 – 9,999 |
| | 10,000 – 49,999 |
| | ≥ 50,000 |
| **Vision assessment method** | Survey |
| | Exam |
| | Interview |
| **Adjustment for potential confounders** | No or minimal (age and/or sex) |
| | Yes (more than age/sex) |
| **Region** | Europe |
| | USA |
| | Asia |
| | Australia |
| | Diverse |
| **Income level** | High income |
| | Low income |

© 2024 Kim CY et al. *JAMA Network Open*.

**eTable 2.** Effect Moderators for Studies on Suicidal Behavior

| Variables | Amount of heterogeneity accounted for (%) | *P* value for test of moderators |
|---|---|---|
| Publication year of the study | 2.42 | 0.22 |
| Main ethnicity of study participants | 0.01 | 0.62 |
| Mean age of study participants | 71.3 | < 0.001 |
| Total sample size | 7.05 | 0.17 |
| Vision assessment method | 1.98 | 0.41 |
| Consideration of potential confounding factors | 0.03 | 0.80 |
| Country where the study was conducted | 0.06 | 0.67 |
| Study conducted in low-income country | 2.44 | 0.35 |

© 2024 Kim CY et al. *JAMA Network Open*.

**eTable 3.** Subgroup Analysis on Study Participants' Mean Age

| Age group | Number of studies | OR | 95% CI | $I^2$ (%) |
|---|---|---|---|---|
| All adults | 11 | 1.71 | 1.38; 2.11 | 54.4 |
| ≥ 65 years | 2 | 6.66 | 2.95; 15.00 | 0.0 |
| ≥ 50 years | 1 | 1.98 | 1.24; 3.14 | - |
| ≥ 40 years | 1 | 1.33 | 1.26; 1.41 | - |
| Adolescents | 2 | 9.85 | 4.39; 22.10 | 84.3 |

OR = odds ratio; CI = confidence interval.

© 2024 Kim CY et al. *JAMA Network Open*.

**eTable 4.** Association Between Visual Impairment and Suicidality Risk After Exclusion of Potential Outliers

| Analysis | OR (95%CI) | *P*-value | $I^2$ (%) |
|---|---|---|---|
| Suicidal behavior | | | |
| Main analysis | 2.49 (1.71-3.63) | < 0.001 | 92.8 (90.0-94.9) |
| Infl. cases removed[*] | 1.83 (1.48-2.28) | < 0.001 | 68.0 (45.2-81.3) |
| Suicide Ideation | | | |
| Main analysis | 2.01 (1.62-2.50) | < 0.001 | 88.8 (84.2-92.0) |
| Infl. cases removed[†] | 1.69 (1.47-1.93) | < 0.001 | 61.8 (37.1-76.8) |
| Death by suicide | | | |
| Main analysis | 1.89 (1.32-2.71) | 0.005 | 73.6 (46.3-87.0) |
| Infl. cases removed[‡] | 1.86 (1.08-3.21) | 0.025 | 68.3 (25.1-86.6) |

[*]Removed as outliers: Lam et al, 2008; Alvarado-Esquivel, 2018.
[†]Removed as outliers: Azzi et al., 2008; Okoro et al., 2021.
[‡]Removed as outliers: Lam et al, 2008; Alvarado-Esquivel, 2018.
OR = odds ratio; CI = confidence interval.

© 2024 Kim CY et al. *JAMA Network Open*.

**eReferences**


1. Jorm AF, Henderson AS, Scott R, Korten AE, Christensen H, Mackinnon AJ. Factors associated with the wish to die in elderly people. *Age Ageing*. 1995;24(5):389-392. Medline:8669340 doi:10.1093/ageing/24.5.389
2. Forsell Y, Jorm AF, Winblad B. Suicidal thoughts and associated factors in an elderly population. *Acta Psychiatr Scand*. 1997;95(2):108-111. Medline:9065674 doi:10.1111/j.1600-0447.1997.tb00382.x
3. Waern M, Rubenowitz E, Runeson B, Skoog I, Wilhelmson K, Allebeck P. Burden of illness and suicide in elderly people: case-control study. *BMJ*. 2002;324(7350):1355. Medline:12052799 doi:10.1136/bmj.324.7350.1355
4. Waern M, Rubenowitz E, Wilhelmson K. Predictors of suicide in the old elderly. *Gerontology*. 2003;49(5):328-334. Medline:12920354 doi:10.1159/000071715
5. Yip PS, Chi I, Chiu H, Chi Wai K, Conwell Y, Caine E. A prevalence study of suicide ideation among older adults in Hong Kong SAR. *Int J Geriatr Psychiatry*. 2003;18(11):1056-1062. Medline:14618559 doi:10.1002/gps.1014
6. Mojon-Azzi SM, Sousa-Poza A, Mojon DS. Impact of low vision on well-being in 10 European countries. *Ophthalmologica*. 2008;222(3):205-212. Medline:18497531 doi:10.1159/000126085
7. Lam BL, Christ SL, Lee DJ, Zheng DD, Arheart KL. Reported visual impairment and risk of suicide: the 1986-1996 National Health Interview Surveys. *Arch Ophthalmol*. 2008;126(7):975-980. Medline:18625946 doi:10.1001/archopht.126.7.975
8. Fässberg MM, Ostling S, Börjesson-Hanson A, Skoog I, Wærn M. Suicidal feelings in the twilight of life: a cross-sectional population-based study of 97-year-olds. *BMJ Open*. 2013;3(2):e002260. Medline:23377994 doi:10.1136/bmjopen-2012-002260
9. Okamura T, Ito K, Morikawa S, Awata S. Suicidal behavior among homeless people in Japan. *Soc Psychiatry Psychiatr Epidemiol*. 2014;49(4):573-582. Medline:24213525 doi:10.1007/s00127-013-0791-y
10. Cho GE, Lim DH, Baek M, Lee H, Kim SJ, Kang SW; Epidemiologic Survey Committee of the Korean Ophthalmological Society. Visual impairment of Korean population: prevalence and impact on mental health. *Invest Ophthalmol Vis Sci*. 2015;56(8):4375-4381. Medline:26176874
11. Kim Y, Kwak Y, Kim JS. The association between suicide ideation and sensory impairment among elderly Koreans. *Aging Ment Health*. 2015;19(7):658-665. Medline:25495960 doi:10.1080/13607863.2014.989812
12. Rim TH, Lee CS, Lee SC, Chung B, Kim SS; Epidemiologic Survey Committee of the Korean Ophthalmological Society. Influence of visual acuity on suicidal ideation, suicide attempts and depression in South Korea. *Br J Ophthalmol*. 2015;99(8):1112-1119. Medline:25733526 doi:10.1136/bjophthalmol-2014-306518
13. Meyer-Rochow VB, Hakko H, Ojamo M, Uusitalo H, Timonen M. Suicides in visually impaired persons: a nation-wide register-linked study from Finland based on thirty years of data. *PLoS One*. 2015;10(10):e0141583. Medline:26509899 doi:10.1371/journal.pone.0141583


© 2024 Kim CY et al. *JAMA Network Open*.

14. Jung KI, Park CK. Mental health status and quality of life in undiagnosed glaucoma patients: a nationwide population-based study. *Medicine (Baltimore)*. 2016;95(19):e3523. Medline:27175648 doi:10.1097/MD.0000000000003523
15. Morton K. Examining diabetes, depression and suicide in middle-aged and older adults. West Virginia University, the Research Repository. 2017. https://researchrepository.wvu.edu/edt/6269
16. Moses T. Suicide attempts among adolescents with self-reported disabilities. *Child Psychiatry Hum Dev*. 2018;49(3):420-433. Medline:29030735
17. Alvarado-Esquivel C. Suicidal ideation and suicide attempts in middle-aged women attending a primary care center: a cross-sectional study in Mexico. *J Clin Med Res*. 2018;10(9):693-699. Medline:30116439 doi:10.14740/jocmr3501w
18. Cosh S, Carrière I, Daien V, Tzourio C, Delcourt C, Helmer C. Sensory loss and suicide ideation in older adults: findings from the Three-City cohort study. *Int Psychogeriatr*. 2019;31(1):139-145. Medline:29798742 doi:10.1017/S104161021800056X
19. Na EJ, Lee H, Myung W, et al. Risks of completed suicide of community individuals with ICD-10 disorders across age groups: a nationwide population-based nested case-control study in South Korea. *Psychiatry Investig*. 2019;16(4):314-324. Medline:31042694 doi:10.30773/pi.2019.02.19
20. Akram B, Batool M. Suicidal behavior among the youth with and without sensory impairment: prevalence and comparison. *Omega (Westport)*. 2020;81(3):393-403. Medline:29860918 doi:10.1177/0030222818779711
21. Park J, Lee OE. Association between vision impairment and suicidal ideation among older adults: results from National Survey on Drug Use and Health. *Disabil Health J*. 2020;13(4):100939. Medline:32417146 doi:10.1016/j.dhjo.2020.100939
22. Smith M, Cui R, Odom JV, Leys MJ, Fiske A. Giving support and suicidal ideation in older adults with vision-related diagnoses. *Clin Gerontol*. 2020;43(1):17-23. Medline:31469341 doi:10.1080/07317115.2019.1659465
23. Khurana M, Shoham N, Cooper C, Pitman AL. Association between sensory impairment and suicidal ideation and attempt: a cross-sectional analysis of nationally representative English household data. *BMJ Open*. 2021;11(2):e043179. Medline:33593780 doi:10.1136/bmjopen-2020-043179
24. Okoro CA, Strine TW, McKnight-Eily L, Verlenden J, Hollis ND. Indicators of poor mental health and stressors during the COVID-19 pandemic, by disability status: a cross-sectional analysis. *Disabil Health J*. 2021;14(4):101110. Medline:33962896 doi:10.1016/j.dhjo.2021.101110
25. Parker M, Duran B, Rhew I, Magarati M, Larimer M, Donovan D. Risk and protective factors associated with moderate and acute suicidal ideation among a national sample of tribal college and university students 2015-2016. *J Rural Health*. 2021;37(3):545-553. Medline:32894614 doi:10.1111/jrh.12510
26. Marlow NM, Xie Z, Tanner R, Jo A, Kirby AV. Association between disability and suicide-related outcomes among US adults. *Am J Prev Med*. 2021;61(6):852-862. Medline:34465506 doi:10.1016/j.amepre.2021.05.035

© 2024 Kim CY et al. *JAMA Network Open*.

27. Marlow NM, Xie Z, Tanner R, et al. Association between functional disability type and suicide-related outcomes among US adults with disabilities in the National Survey on Drug Use and Health, 2015-2019. *J Psychiatr Res*. 2022;153:213-222. Medline:35841817 doi:10.1016/j.jpsychires.2022.07.014
28. Smith L, Shin JI, Barnett Y, et al. Association of objective visual impairment with suicidal ideation and suicide attempts among adults aged ≥50 years in low/middle-income countries. *Br J Ophthalmol*. 2022;106(11):1610-1616. Medline:33931392 doi:10.1136/bjophthalmol-2021-318864
29. Lee OE, Park D, Park J. Association of vision impairment with suicide ideation, plans, and attempts among adults in the United States. *J Clin Psychol*. 2022;78(11):2197-2213. Medline:36017683 doi:10.1002/jclp.23437
30. Ha A, Kim SH, Kang G, Yoon HJ, Kim YK. Association between sight-threatening eye diseases and death by suicide in South Korea: a nationwide population-based cohort study. *Ophthalmology*. 2023;130(8):804-811. Medline:37001591 doi:10.1016/j.ophtha.2023.03.018
31. Sung C, Chung CH, Lin FH, et al. A population-based cohort study of the association between visual loss and risk of suicide and mental illness in Taiwan. *Healthcare (Basel)*. 2023;11(10):1462. Medline:37239748 doi:10.3390/healthcare11101462

© 2024 Kim CY et al. *JAMA Network Open*.
