# Supplementary material for: Visual Impairment and Suicide Risk: A Systematic Review and Meta-Analysis
Source: JAMA Netw Open. 2024 Apr 17;7(4):e247026. doi: 10.1001/jamanetworkopen.2024.7026 (PMC11024775; doi:10.1001/jamanetworkopen.2024.7026)
Supplement: Supplement 2. — Data Sharing Statement [file jamanetwopen-e247026-s002.pdf]

Data Sharing Statement

Kim. Visual Impairment and Suicide Risk. *JAMA Netw Open*. Published April 17, 2024.
doi:10.1001/jamanetworkopen.2024.7026

Data
Data available: No

Additional Information
Explanation for why data not available: This manuscript does not include original data. Data are extracted from literature and are publicly available.
